# Impact of ploidy level on the distribution of *Pokey* element insertions in the *Daphnia pulex* complex

**DOI:** 10.1186/1759-8753-5-1

**Published:** 2014-01-02

**Authors:** Roland Vergilino, Shannon HC Eagle, Teresa J Crease, France Dufresne

**Affiliations:** 1 Département de Biologie, Chimie et Géographie, Université du Québec à Rimouski, Rimouski, Québec G5L 3A1, Canada; 2 Department of Integrative Biology, University of Guelph, Guelph, Ontario N1G 2W1, Canada; 3 Centre d’Études Nordiques, Université Laval, Québec G1V 0A6, Canada

**Keywords:** *Daphnia pulex*, Hybrids, Insertion site polymorphism, Load, *Pokey*, Polyploids, Transposable element

## Abstract

**Background:**

Transposable elements (TEs) play a major role in genome evolution. Their capacity to move and/or multiply in the genome of their host may have profound impacts on phenotypes and dramatic consequences on genome structure. The population dynamics and distribution of TEs are influenced by their mode of transposition, the availability of niches in host genomes, and host population dynamics. Theories predict an increase in the number of TE insertions following hybridization or polyploidization. Evolution of TEs in hybrids and polyploids has mostly been studied in plants; few studies have examined the impacts of hybridization and/or polyploidization on TEs in animals. Hybrids and polyploids have arisen multiple times in the *Daphnia pulex* complex and are thought to reproduce by obligate parthenogenesis. Our study examines the effects of ploidy level on polymorphism and number of *Pokey* element insertions in diploid and polyploid hybrid isolates from the *Daphnia pulex* complex.

**Results:**

The polymorphism of *Pokey* insertion sites did not depend solely on either the ploidy level or the genetic background of their host; therefore, it may be the result of interactions between these parameters and other parameters such as *Pokey* activity, selection and/or drift. No significant effect of ploidy level was found on the number of *Pokey* insertions using TE display and qPCR. However, the load of *Pokey* insertion sites and the number of unique insertion sites were slightly (but not significantly) higher in polyploids than in diploids.

**Conclusions:**

These results suggest a lack of increase in the number of *Pokey* insertions following polyploidization but higher availability of *Pokey* insertion sites in polyploids than in diploids. Compared to previous TE display and qPCR results, the load of *Pokey* insertions in hybrid diploids was higher than in non-hybrid sexual and asexual diploids, which suggests an increase in the density of *Pokey* insertions following hybridization.

## Background

Transposable elements (TEs) are genetic components that are able to move and multiply within and between genomes. They are found in the genomes of almost all living organisms [1], although there are exceptions in endosymbiont organisms [2]. There is large variation in the proportion of TEs across genomes [3]. TE populations are impacted by host population dynamics, such as effective population size, mode of reproduction, hybridization, and polyploidization [4-6]. A decrease in effective population size of the host [7] or an increase in its level of selfing [8] are expected to lead to an increase in the density of TE insertions. The mode of reproduction of the host also has a substantial impact on the dynamics and density of TE insertions in the genome [9-12]. For example, TEs may spread via recombination and out-crossing in sexual populations [11], whereas the spread of TEs among lineages is prevented in asexual populations except by horizontal transmission [13,14]. Empirical studies have tested and are generally in accordance with the theoretical prediction that the genomes of sexual organisms will contain a higher number of TE insertions compared to asexual ones [10,12-15]. Hybridization and polyploidization, which play a significant role in the diversification of plants and animals [16-18], might also have an impact on the load and insertion site polymorphism of TEs. Activation of TEs has been observed in hybrid genomes [19-22] (however, there are contradictory results in hybrid sunflowers [23]), and polyploidization may lead to an increase in the density of TE insertions [24,25], although there are contradictory results in allopolyploid plants [26]. Bursts of TE activity are thought to have a substantial impact on genome rearrangement [27] and may lead to phenotypic diversification in hybrids and polyploids [24,28]. Many studies have explored the effects of hybridization and polyploidization on TE dynamics in plants [22,23,26,28-34], but few studies have focused on these effects in animals, with the exception of studies in carp [35], Drosophila [36] and wallaby [20], all of which have been reviewed [37]. Studying the dynamics of TEs in hybrids and polyploids may provide insight on the evolution of their genomes and their propensity to adapt to various environments.

The *Daphnia pulex* (*D. pulex*) species complex has been intensively studied due to its dominance in freshwater habitats in North America and its variation in reproductive mode and ploidy level. *Daphnia* usually reproduce by cyclic parthenogenesis, which is clonal reproduction interrupted by bouts of sexual reproduction. However, some lineages reproduce by obligate parthenogenesis (i.e., without any sexual reproduction) [38-42]. The *D. pulex* complex includes numerous lineages that have been distinguished on the basis of morphological, ecological, and genetic data [43-47]. Analyses of mitochondrial DNA variation have revealed the presence of three major groups in this complex. The pulicaria group consists of five different lineages; North American *D. pulicaria* (with three sublineages, Eastern *D. pulicaria*, Western *D. pulicaria* and Polar *D. pulicaria*), *D. pulex, D. melanica*, *D. middendorffiana sensu stricto*, and *D. arenata*, an endemic species inhabiting Oregon ponds [43,48]. The tenebrosa group includes two lineages, European *D. pulicaria* and *D. tenebrosa*[43]. The third group includes European *D. pulex*. Mitochondrial lineages in the pulicaria group may have diverged during the Pleistocene (between 1.2 and 2.2 million years ago) [43,49,50] while the pulicaria and tenebrosa groups seem to have diverged during the Pliocene (around 3 million years ago) [43]. Relationships between lineages based on nuclear genes are less clear and may be confounded by incomplete lineage sorting and a highly reticulate history [45,51,52]. In North America, two lineages, *D. pulex* and *D. pulicaria* (considered to be ecological species), are dominant in freshwater habitats. They are morphologically similar but ecologically distinct [44], although they hybridize in nature [42,53-55]. *D. pulex* and F1 hybrids are usually found in fishless shallow ponds whereas *D. pulicaria* inhabits lakes. Variation in the *Lactate dehydrogenase* gene (*Ldh*) is diagnostic [54,55]; *D. pulex* is fixed for the S allele whereas the F allele is fixed in *D. pulicaria*[55]. Diploid hybrids of these two lineages possess an SF genotype at the *Ldh* locus, always have *D. pulex* mitochondrial genomes, and have been found to reproduce by obligate parthenogenesis in nature [42,56], although laboratory-produced hybrids may be able to reproduce by cyclical parthenogenesis [56,57]. It has been suggested that hybridization may play a role in the spread of meiosis suppressing genetic elements in the obligate parthenogenetic populations of *D. pulex* with SS *Ldh* genotypes via introgression [58,59].

Polyploidy has evolved repeatedly in the *D. pulex* complex [49,60-62] and shows a geographical pattern [49,62-66]. Polyploid populations are obligate parthenogens and are found at high latitudes and altitudes, and diploid populations (hybrid or not) are prevalent in temperate regions [47,60,62]. A polyphyletic assemblage of polyploids collectively known as *D. middendorffiana* (and which we term *D. middendorffiana sensu lato* in this study) is thought to have arisen from hybridization between *D. pulex* males and *D. pulicaria* females, or females of another species which no longer exists as a cyclic parthenogen [49,60,61]. Other polyploids are thought to have arisen from crosses between *D. pulex* females and *D. pulicaria* males and are encountered in the Northeast of Quebec and in Ontario (Canada) [45,67]. Moreover, *D. tenebrosa*, a circumarctic species [62], includes both diploids and polyploids [67], but the hybrid nature of the polyploids in this species is still unclear [45]. A study using microsatellite data, flow cytometry, and mitochondrial sequences has shown that most polyploids of the *D. pulex* complex are triploids, although some tetraploids have also been observed [67].

The *D. pulex* genome of one cyclically parthenogenetic isolate from Oregon has been sequenced [68], and numerous class II TEs have been identified in it [9]. Previous studies have reported that the class II TE load is lower in the genomes of obligate compared to cyclical parthenogenetic *D. pulex* lineages [10,15], as theoretically predicted if sexual reproduction helps TEs to spread [11]. *Pokey*, a class II TE from the *piggyBac* superfamily, has been extensively studied in diploid populations of *Daphnia*. It inserts in the tandemly repeated rRNA genes [69] and in other parts of the genome [15,70]. Based on patterns of polymorphism in *Pokey* insertion sites observed among natural populations, previous studies have suggested that *Pokey* may be active in cyclically parthenogenetic populations of *D. pulex* but not in obligate parthenogens [15,70]. The diversity, and potentially the activity, of *Pokey* in rRNA genes is greatly influenced by recombination events, especially in hybrids [71]. The *D. pulex* complex and *Pokey* represent an interesting model to study the effect of hybridization and polyploidization on the evolution and dynamics of a class II TEs *in natura.*

The aim of this study is to compare the polymorphism of *Pokey* insertion sites between diploid and polyploid hybrid genomes in obligately parthenogenetic isolates of the *D. pulex* complex. If *Pokey* was not active during and following hybridization events, the similarity between *Pokey* insertion profiles should be congruent with host evolutionary relationships. To test this prediction, the polymorphism of *Pokey* insertion site profiles was compared with the ploidy level and the genetic similarity of the hosts determined using microsatellite multilocus genotypes. Moreover, we test the prediction that the number of *Pokey* per haploid genome (hereafter called density) is similar in polyploid and diploid hybrids using two complementary techniques, TE display and quantitative PCR (qPCR). TE display allows us to compare the diversity of *Pokey* insertion sites in polyploid and diploid isolates. This technique also provides an estimate of the number of *Pokey* insertion sites (*Pokey* load) but it cannot distinguish between homozygosity and heterozygosity at a particular site. Conversely, qPCR allows us to estimate the total number of *Pokey* insertions per haploid genome (*Pokey* density) regardless of location, including those that occur in rDNA, which appear as a single peak in a TE display analysis. A higher density of *Pokey* insertions per haploid genome in polyploids than in diploids may be evidence of an increase in *Pokey* activity after polyploidization.

## Methods

### *Daphnia* samples

In the laboratory, we established parthenogenetic lines of *Daphnia* (hereafter called isolates) from 27 individual obligately parthenogenetic females (14 diploid hybrids and 13 polyploid hybrids) sampled from ponds in North America between 2004 and 2008 (Additional file 1). *Daphnia* were sampled from ponds accessed via public roadsides or on private land with the permission of the land owner. No specific permissions are required to sample *Daphnia* as they are not endangered or protected species. The lines were cultured using standard techniques [72]. The isolates represent six mitochondrial lineages (*D. pulex*, Polar *D. pulicaria*, Western *D. pulicaria*, Eastern *D. pulicaria*, *D. middendorffiana sensu stricto*, and *D. tenebrosa*). Due to the geographical polyploidy pattern, all the polyploids come from two subarctic regions (Churchill, MB, Canada and Kuujjuarapik, QC, Canada), although the diploids come from both temperate and subarctic regions (Additional file 1). For each isolate, genomic DNA from 10 to 30 individuals, weighing approximately 100 mg (wet weight), was extracted using the DNeasy Tissue kit (QIAGEN Inc., Mississauga, ON, Canada) according to the supplier’s protocol. Origin of the putative parental species of each isolate (Additional file 1) was determined by combining information on morphology, haplotype of the mitochondrial ND5 gene, and genotype at the nuclear *Ldh* gene [45]. Ploidy levels were previously assessed using nine microsatellite loci and flow cytometry [45].

### TE display

We used a PCR-based approach called TE display [73], which generates dominant AFLP-like markers, to test the effect of ploidy level on insertion site polymorphism, and on the load of *Pokey* insertions in the genomes of 14 diploid and 13 polyploid isolates. We followed a modified version of the TE display protocol of Valizadeh and Crease [15] that involves digestion of genomic DNA using the restriction enzyme BfaI followed by ligation of BfaI linkers and two rounds of PCR amplification using a *Pokey*-specific forward primer (Additional file 2). The ligated DNA was used as a template for a primary (pre-selective) PCR with the primer Pok6456F, located near the 3′ end of *Pokey*, and the primer BfaI-R that anneals to the BfaI linker sequence followed by a secondary (selective) PCR using fluorescent labeled primer Pok6464F and the primer BfaI-R (Table 1). Our TE display protocol, unlike that of Valizadeh and Crease [15], used an annealing temperature of 50°C instead of 55°C for both the primary and secondary PCR. This allows amplification of *Pokey* insertions in *Daphnia* species with genomes that are divergent from *D. pulex*. Only fragments ≥160 bp were included in our analyses and primary PCR were repeated three times in each individual followed by a secondary PCR on the product of each primary reaction to ensure that *Pokey* profiles were reproducible and to remove possible artefacts from our analysis (Additional file 2).

**Table 1:** TE display and qPCR primers and linkers used in this study

**Purpose**	**Annealing temperature**	**Primer name**	**Sequence (Dye)**	**Percent amplification efficiency**	**Amplicon size**
Linkers for TED	/	BfaI Linker F	5′-TACTCAGGACTCAT	/	/
		BfaI Linker R	5′-GACGATGAGTCCTGAG		
Primary PCR for TED	50°C/55°C	Pok6456F	5′-GACAACGGTGGCCGAAACGCGG	/	/
		BfaIR	5′-GACGATGAGTCCTGAGTAG		
Secondary PCR for TED	50°C/55°C	Pok6464F	5′-TGGCCAAAACACGGTTTGGCCG (HEX)	/	/
		BfaIR	5′-GACGATGAGTCCTGAGTAG		
18S genes for qPCR	60°C	18S1864F	5′-CCGCGTGACAGTGAGCAATA	0.9556	50
		18S1913R	5′-CCCAGGACATCTAAGGGCATC		
28S genes for qPCR	60°C	28S3054F	5′-GGTAGCCAAATGCCTCGTCA	0.9246	150
		28S3204R	5′-GAGTCAAGCTCAACAGGGTCTTCTTTCCC		
Total *Pokey* for qPCR	60°C	Pok6456F	5′-GACAACGGTGGCCGAAACGCGG	0.9136	122
		Pok6578R	5′-GATGGTCGGATTCGATTGAATGCTCG		
*Pokey* in rDNA for qPCR	60°C	Pok6456F	5′-GACAACGGTGGCCGAAACGCGG	0.8957	192
		28S3104R	5′-GTTAATCCATTCGTGCGCG		
*Tif* for qPCR	60°C	TIF392F	5′-GACATCATCCTGGTTGGCCT	0.9493	50
		TIF442R	5′-AACGTCAGCCTTGGCATCTT		
*Gtp* for qPCR	60°C	GTP385R	5′-TATTCAGCATGGAGAGACGGC	0.9369	50
		GTP435R	5′-GATGTCGACTGACGCTGGAA		

### Comparison of genetic distance based on *Pokey* profiles, microsatellites and the *ND5* gene

We used the results of TE display to generate a binary matrix of presence (1) or absence (0) of peaks, which represents the *Pokey* insertion profile (Additional file 3). We then generated a matrix of Jaccard distance estimates from the *Pokey* profiles. The Jaccard distance was chosen because it does not use shared absence of an allele as a shared characteristic [74]. A distance matrix was also calculated for each locus of the microsatellite dataset previously obtained for our isolates [45] using a modified version of the Bruvo distance [75], implemented in the PolySat package [76] using the R software [77]. The Bruvo distance allowed us to estimate relationships of mixed-ploidy level genotypes using co-dominant markers. The Bruvo distance takes into account stepwise mutation models between alleles. In the non-modified version (equation 2 in [75]), the algorithm adds “virtual allele” with an “infinite” value to lowest ploidy-level genotypes to compare them to the highest ploidy-level genotypes. This may lead to group artificially genotypes with the same ploidy level [78]. Thus, we used a modified version of the Bruvo distance (Bruvo2.distance implemented in PolySat set with the parameters add = TRUE and loss = TRUE) that allows genome “addition” and “loss”, simulating gene addition by polyploidization but also possible gene loss via diploidization. This modified version of the Bruvo distance does not lead to artificially grouping genotypes with the same ploidy level altogether. In addition, we generated a matrix of sequence divergence between *ND5* sequences from previous studies by Vergilino et al. [45,67] (Table 1 for Genbank accession number) from our isolates using the maximum composite likelihood model implemented in MEGA5.1 [79].

To determine if the *Pokey* insertion sites profiles differed depending on the genetic background and ploidy level between isolates, a principal coordinate analysis (PCoA) [80] and a K-means cluster analysis were conducted to represent affinities between the different *Pokey* insertion profiles or multilocus microsatellite genotypes using R software version 2.15.2 [77,81]. Each PCoA was constructed using the pco module of the labdsv library in the R software on the Jaccard distance matrix for the *Pokey* profiles and the modified Bruvo distance matrix for the microsatellites after transforming these distance matrices in Euclidean distances [82]. The K-means analyses were conducted on binary matrices representing either the *Pokey* profiles or microsatellite genotypes (transformed into a binary matrix), and the number of clusters for each analysis was set using an iterative method, CascadeKM with the calinski criterion [83], implemented in the vegan package available with R software. We also performed a Mantel test according to Legendre and Legendre (section 10.5 in [74]) using the Pearson method with 10,000 replicates (package vegan in R software) to compare the Jaccard distance matrix based on *Pokey* insertion profiles with both the Bruvo distance matrix based on microsatellite data and the distance matrix based on *ND5* mitochondrial haplotypes.

To test the hypothesis that the load of *Pokey* insertions increases with ploidy level, we compared the number of *Pokey* insertion sites estimated by TE display to the ploidy level after taking into account the heterozygosity of the isolates. Heterozygosity was weighted by ploidy level and was calculated from the variability of nine microsatellite loci [45]. Theoretically, polyploids may arise from independent hybridization events and those with different parental genomes may possess a higher diversity of TE insertion sites than polyploids with similar parental genomes due to increased probability of homozygosity of some TE insertions in the latter case. If we do not account for the different genomes that form polyploids, then we may overestimate the effect of ploidy level since the number of TE insertions could be more strongly correlated with heterozygosity level than ploidy level. Thus, we introduce a ploidy-weighted heterozygosity index (*H*_*pl*_) for a comparison between diploids and triploids, which takes into account the ploidy level of each genotype such that:

$$H_{\mathrm{pl}}=\frac{n_{3}+0.5*n_{2}}{n_{L}}$$

where *n*_*L*_ is the total number of microsatellite loci analyzed (9), *n*_*3*_ is the number of loci with 3 different alleles, and *n*_*2*_ is the number of loci with only 2 different alleles. Genotypes that are homozygous for all microsatellite loci have an *H*_*pl*_ of 0, diploid isolates that are heterozygous for every locus and triploid isolates with two different alleles at every locus have an *H*_*pl*_ of 0.5, and triploid isolates that have three different alleles at every locus have an *H*_*pl*_ of 1. Therefore, triploids with low *H*_*pl*_ values (under 0.5) can be compared to diploid hybrids. To disentangle the effect of adding different genomes from the effect of increased ploidy level, we performed an ANCOVA (Analysis of Covariance) using R software [77] with the number of *Pokey* insertions as the dependent variable and the ploidy level and *H*_*pl*_ as the independent variables. The number of singletons (i.e., *Pokey* insertion sites encountered in only one isolate) between diploid and polyploid isolates was compared using a Fisher exact test performed on a 2 × 2 contingency table similar to the approach of Wright et al. [73].

Direct comparison of our results to those obtained on cyclic and obligate non-hybrid diploid populations previously studied by Valizadeh and Crease [15] was not possible as these authors used a higher annealing temperature (55°C instead of 50°C). Therefore, we performed additional TE display assays on six diploid hybrid and six polyploid hybrid isolates using the 55°C annealing temperature of Valizadeh and Crease [15].

### qPCR assays

Because TE display generates dominant markers, it provides more information about the polymorphism of *Pokey* insertion sites than their density within the genome. This difference may be significant especially if a significant proportion of *Pokey* insertions are homozygous, which may be possible in polyploids [24,25]. Therefore, to help resolve this problem, the number of *Pokey* insertions per haploid genome was estimated using qPCR. We performed qPCR assays on *Pokey* inserted in 28S rRNA genes (r*Pokey*) and in the entire genome (t*Pokey*) of 9 diploid and 10 polyploid isolates as described by Eagle and Crease [84]. We also estimated the number of 18S and 28S rRNA genes as the number of r*Pokey* may be correlated to the number of rRNA genes [84,85]. Briefly, we used the ΔC_T_ qPCR method as described in Eagle and Crease [84] (Additional file 2) to estimate the density of multicopy genes (18S, 28S, t*Pokey*, r*Pokey*) relative to two single-copy genes (Table 1); *Tif*, a transcription initiation factor and *Gtp*, a member of the RAB subfamily of small GTPases. Assuming that diploids have two copies and triploids have three copies of these two genes, these estimates correspond to the haploid number of multicopy genes in each genome. Reaction conditions were run in triplicate as described in Eagle and Crease [84] (Additional file 2). The mean haploid copy number, rounded to the nearest 0.5 for diploids and 0.34 for triploids, and standard deviations were calculated for each multicopy gene in each isolate. The number of *Pokey* insertions outside 28S rRNA genes per haploid genome (g*Pokey*) was calculated as [t*Pokey* number – r*Pokey* number].

We used modules available in the R software package to perform correlation and regression analyses between the haploid number of 18S rRNA genes, 28S rRNA genes, r*Pokey*, and g*Pokey* in diploids and polyploids. Levene’s tests (equality of variances) and Student’s *t*-tests (equality of means) were used to test for possible significant differences in 18S, 28S, r*Pokey*, and g*Pokey* haploid numbers between diploids and polyploids. The sequential Bonferroni technique proposed by Rice [86] was used to adjust the significance level (0.05) for the multiple Student’s *t*-tests comparing 18S and 28S number within isolates.

Under the assumption that the same g*Pokey* elements are amplified using qPCR and TE display techniques, we can estimate the average heterozygosity for these elements in diploids by using:

$$H_{\mathrm{Pokey}}=\frac{2\left(n_{\mathrm{TED}-1}-n_{\mathrm{gPokey}}\right)}{n_{\mathrm{TED}-1}}$$

where *n*_*TED-1*_ represents the number of different *Pokey* insertion sites estimated by TE display minus the peak representing r*Pokey*, and *n*_*gPokey*_ is the haploid number of g*Pokey* estimated by qPCR. However, due to partial heterozygosity in triploids, we were not able to calculate their exact heterozygosity level. The ratio (k*n_*gPokey*_)/*n*_*TED-1*_, where *k* is the ploidy level of the isolate, allows us to evaluate if TE display and/or qPCR techniques underestimate or overestimate *Pokey* insertions. If every g*Pokey* insertion is in a heterozygous state, this ratio will be 1. If the ratio is below 1, qPCR underestimates or TE display overestimates the number of *Pokey* insertions. If all insertions are in a homozygous state, the ratio equals 2 for diploids and 3 for triploids. If the ratio is greater than 2 or 3 in diploids or triploids, respectively, qPCR overestimates or TE display underestimates the number of *Pokey* insertions.

## Results

### Polymorphism of *Pokey* insertion site profiles

Using the TE display technique, the average number of *Pokey* insertion sites in 14 diploid isolates was 16.64 (±4.94), with values from 6 to 26, whereas the average number of *Pokey* insertion sites in 13 polyploid isolates was 19.00 (±4.36), with values from 12 to 27. The two means are not significantly different (Student t-test, *t* = −1.3105, *df* = 25, *P* = 0.202; Table 2; Additional file 1). Overall, 88 different *Pokey* insertion sites were detected (Additional file 3). Such polymorphism allowed us to analyze the similarity between isolates based on these *Pokey* profiles and on microsatellite genotypes using PCoA. In addition, the *Pokey* profiles and the genetic similarity of the hosts based on microsatellite loci and mitochondrial haplotypes were compared using Mantel tests.

**Table 2:** **Summary of TE display and qPCR analyses of *Pokey *** **number in diploid and polyploid isolates in the *Daphnia pulex *** **complex**

**Isolates**	**TE display**		**qPCR**					
	**Anneal at 50°C**	**Anneal at 55°C**	**18S genes**	**28S genes**	**Total Pokey**	**rDNA Pokey**	**Genome Pokey**	**Tif:Gtp**
							**(Total-rDNA)**	
Diploids with known hybrid status	19.09 ± 3.99 [11]	13.83 ± 4.22 [6]	293.25 ± 111.19 [8]	486.25 ± 201.36 [8]	17.81 ± 4.35 [8]	5.19 ± 5.03 [8]	12.63 ± 5.04 [8]	0.91 ± 0.06 [8]
Polyploids with known hybrid status	21.50 ± 3.54 [10]	16.33 ± 1.75 [6]	214.57 ± 62.34 [7]	346.67 ± 113.86 [7]	15.57 ± 3.43 [7]	3.10 ± 1.07 [7]	12.48 ± 3.20 [7]	0.92 ± 0.07 [7]
Total diploids	16.64 ± 4.94 [14]	12.71 ± 4.86 [7]	292.78 ± 104.02 [9]	488.28 ± 188.45 [9]	16.67 ± 5.32 [9]	5.05 ± 4.72 [9]	11.61 ± 5.61 [9]	0.90 ± 0.06 [9]
Total polyploids	19.00 ± 4.36 [13]	14.44 ± 3.17 [9]	248.03 ± 75.21 [10]	398.10 ± 127.42 [10]	14.33 ± 3.45 [10]	3.77 ± 1.40 [10]	10.57 ± 4.03 [10]	0.92 ± 0.08 [10]

Results from qPCR are average ± standard deviation of *Pokey* inserts per haploid genome whereas results from TE display are for whole genomes. Numbers in brackets are the number of isolates tested. *Tif:Gtp* ratio is the number of *Tif* relative to the number of *Gtp* single copy reference genes.

**Figure 1:**
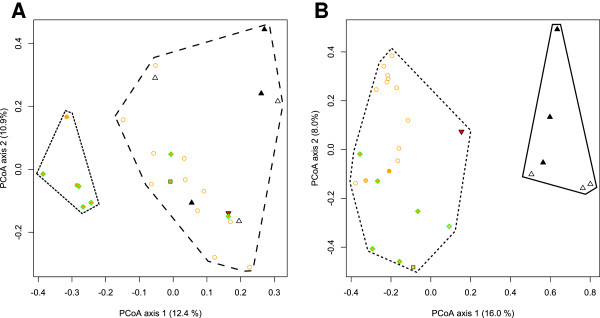
**Principal Coordinate Analyses of Jaccard distance matrix of *Pokey *profiles and Bruvo distance matrix of microsatellite diversity in diploid and polyploid isolates of the *Daphnia pulex *complex. (A) ***Pokey* profiles generated using TE display; **(B)** Microsatellite genotypes determined by Vergilino et al. [45]. The first two axes are represented in each graph. Empty symbols are diploids and solid symbols are polyploids. Empty orange circles: diploid hybrids with *D. pulex* mitochondrial haplotypes; solid orange circles: triploid hybrids with *D. pulex* mitochondrial haplotypes; solid square: *D. middendorffiana sensu stricto*; empty black triangles: diploid *D. tenebrosa*; solid black triangles: triploid *D. tenebrosa*; solid red triangle: introgressed *D. tenebrosa* with a *D. pulex* nuclear genome; solid green diamond filled with orange: triploid hybrids with *D. pulicaria* mitochondrial haplotypes.

The first two axes of the PCoA accounted for 23.3%, with 12.4% for axis 1 and 10.9% for axis 2, of the total variability in *Pokey* profiles (Figure 1A; Additional file 4A). For the host genetic backgrounds, the first two axes of the PCoA accounted for 24.0%, with 16.0% for axis 1 and 8.0% for axis 2, of the total microsatellite variability (Figure 1B and Additional file 4B). *Pokey* profiles and their host microsatellite genotypes ordinate differently in each PCoA according to the two first axes (Figure 1). Although according to the PCoA on the host genetic background, axis 2 differentiates the hybrids with *D. pulex* mitochondria from the *D. middendorffiana sensu lato* (Figure 1B); it is axis 1 that differentiates these isolates according the PCoA from *Pokey* profiles. The second PCoA axis of the *Pokey* profiles does not show clear differentiation pattern according to the mitochondrial haplotype or the ploidy level (Figure 1A). The K-means analyses of both *Pokey* profiles and microsatellite genotypes separate the isolates into two clusters (K = 2, Calinski criterion), but each cluster represents a different set of isolates in the two datasets (Figure 1). One cluster based on *Pokey* profiles contains only polyploid isolates from Kuujjuarappik (Quebec) and Churchill (Manitoba) possessing *D. pulex* (PX3-QC-1 and PX3-QC-2), Eastern *D. pulicaria* (PC3-QC-1, PC3-QC-2 and PC3-QC-3) or Western *D. pulicaria* (PC3-MB-6) mitochondrial haplotypes. The second cluster based on *Pokey* profiles contains diploid hybrids with *D. pulex* mitochondrial haplotypes, *D. tenebrosa* isolates, and polyploids with Polar *D. pulicaria* (PC3-MB-4 and PC3-MB-5) or *D. middendorffiana sensu stricto* (MI3-MB-2) mitochondrial haplotypes. In contrast, one cluster based on microsatellites contains all the *D. tenebrosa* isolates (both diploids and polyploids) while the other cluster contains all the other isolates. Despite these differences, the distance matrices of *Pokey* insertion site profiles and both microsatellite genotype and mitochondrial haplotype datasets are partially correlated according to Mantel tests (Mantel test; *r* = 0.3957, *P* = 0.0001 and r = 0.3047, *P* = 0.003, respectively). The third axis of the PCoA constructed from *Pokey* profiles (9.3%, Additional file 4) may explain why this distance matrix is partially correlated with the distance matrix based on microsatellite genotypes. *Pokey* profiles from *D. tenebrosa* isolates ordinate together according to axis 3 in the PCoA of *Pokey* (Additional file 4A) as they ordinate altogether according to axis 1 in the PCoA of microsatellites (Additional file 4B).

**Figure 2:**
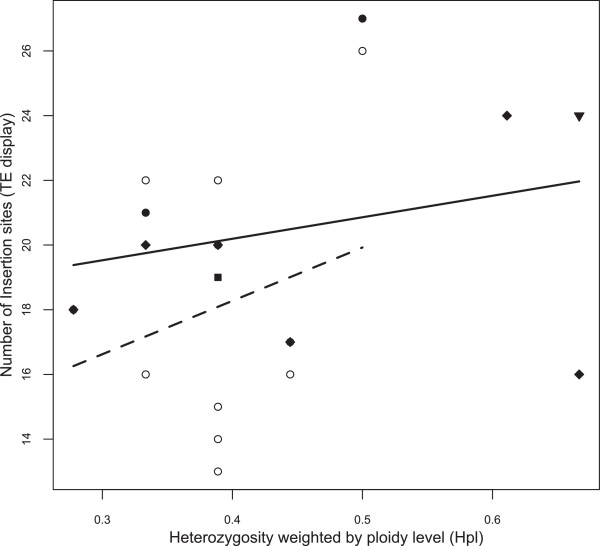
**Relationship between *Pokey *insertion site number outside rDNA estimated using TE display and ploidy-weighted heterozygosity (*H***_***pl***_**) in diploid and polyploid hybrids of the *Daphnia pulex *complex.** Empty circles: diploid hybrids with *D. pulex* mitochondrial haplotypes; solid circles: triploid hybrids with *D. pulex* mitochondrial haplotypes; solid square: *D. middendorffiana sensu stricto*; solid triangle: introgressed *D. tenebrosa* with a *D. pulex* nuclear genome; solid diamond: triploid hybrids with *D. pulicaria* mitochondrial haplotypes. Dashed and solid lines are linear regressions estimated from the data.

As the purpose of our study was to test the effect of ploidy level and not the effect of hybridization, six isolates with *D. tenebrosa* mitochondrial haplotypes and unknown hybrid origin (TE2-MB-1, TE2-MB-2, TE2-MB-3, TE3-MB-1, TE3-MB-2 and TE3-MB-3; Additional file 1) were excluded from the ANCOVA analysis. Excluding these isolates, 88 different *Pokey* insertion sites were detected using TE display (Additional file 3). The mode of reproduction is not a confounding effect in this analysis as both diploid and polyploid hybrids are obligate parthenogens. The mean number of *Pokey* insertion sites in diploid hybrid isolates is 18.09 (±3.99), with values from 13 to 26, whereas the mean number of *Pokey* insertion sites in polyploid isolates is 20.60 (±3.47), with values from 16 to 27. These means are not significantly different (Student *t*-test, *t* = −1.531, *df* = 19, *P* = 0.1423; Table 2; Additional file 1). Correlations between average number of *Pokey* insertion sites and the average heterozygosity weighted by host ploidy level (*H*_*pl*_) were positive but not significant (Table 3). *H*_*pl*_, ploidy level and *H*_*pl*_*:ploidy* interaction had no significant effect on the number of *Pokey* insertion sites (ANCOVA, *F* = 2.132, *df* = 1, *P* = 0.162; *F* = 1.162, *df* = 1, *P* = 0.296 and *F* = 0.205, *df* = 1, *P* = 0.657, respectively; Figure 2; Additional file 5). The 11 diploid hybrid isolates displayed 65 of the 88 *Pokey* insertion sites, of which 8 (12.3%) are singletons. The 10 polyploid hybrid isolates displayed 68 of the 88 *Pokey* insertion sites, of which 14 (20.6%) are singletons. Twenty-one *Pokey* insertions sites were only sampled in polyploid hybrids while 18 were only sampled in diploid hybrids, and this difference is not statistically significant (Fisher exact test, *P* = 0.8559). The difference in the number of singletons between diploid and polyploid hybrids is not statistically significant (Fisher exact test, *P* = 0.3579). The number of *Pokey* insertion sites observed with TE display using an annealing temperature of 55°C, as in Valizadeh and Crease [15], was lower than that observed using an annealing temperature of 50°C (12.71 ±4.86 *vs.* 16.75 ±6.07, paired Student’s *t*-test, *t* = 3.9506, *df* = 6, *P* = 0.008 for diploids and 14.44 ±3.17 *vs.* 18.78 ±4.86; paired Student’s *t*-test, *t* = 5.3072, *df* = 8, *P* = 0.0007 for polyploids; Additional file 1). The number of *Pokey* insertion sites was about two times lower with an annealing temperature of 55°C in some isolates. For example, 13 sites were detected using an annealing temperature of 50°C but only 6 were detected using 55°C in the diploid hybrid PX2-MB-1 (Additional file 1). Although we are aware that artefacts can be produced during the TE display process, these differences do not seem to be due to a higher frequency of artefacts at 50°C than in 55°C as most artifacts were encountered using both annealing temperatures and were excluded from analysis as indicated in the Methods section.

**Table 3:** **Correlations between *Pokey *** **and rRNA gene number in diploid and polyploid *Daphnia *** **from North America**

**Cytotypes**	**X-axis**	**Y-axis**	**Slope**	**y-intercept**	**R**^**2**^	**P-value**	**Figure**
All diploids	18S	28S	1.8038	−39.8411	0.9901	1.76 e-08*	AF6^8^
All triploids	18S	28S	1.6627	−14.3154	0.9586	5.07 e-07*	AF6
Diploid hybrids	*Hpl*^1^	*Pokey* (TED)^2^	16.500	11.674	0.0634	0.4549	2
	18S	r*Pokey*^3^	−0.0146	9.4540	0.1035	0.4371	AF7
	g*Pokey*^4^	r*Pokey*	−06251	13.0792	0.3927	0.0964	AF8
	*Pokey*-1 (TED)^5^	Total g*Pokey*^6^	1.5329	−1.7681	0.4950	0.0515	3
Diploid hybrids - PX2-MB-1	18S	r*Pokey*	0.0147	−1.07571	0.3979	0.1287	AF7
	g*Pokey*	r*Pokey*	−0.3350	8.0216	0.5605	0.0528	AF8
Diploid hybrids – (PX2-QC-9, PX2-MI-7)	*Pokey*-1 (TED)	Total g*Pokey*	1.5741	−2.7883	0.7383	0.0283*	-
	*Pokey*-1 (TED)	*H*_*Pokey*_^7^	−0.0088	0.7623	0.0376	0.7130	AF9
	*Hpl*	*H*_*Pokey*_	−3.2220	1.8820	0.6592	0.0497*	AF10
Triploid hybrids	*Hpl*	*Pokey* (TED)	6.6490	17.534	0.0756	0.4418	2
	18S	r*Pokey*	0.0085	1.2673	0.2492	0.2541	AF7
	g*Pokey*	r*Pokey*	0.0201	2.8478	0.0036	0.8978	AF8
	*Pokey*-1 (TED)	Total g*Pokey*	−1.6300	1.0290	0.3342	0.1740	3
Triploid tenebrosa	*Pokey*-1 (TED)	Total g*Pokey*	−0.1923	20.7692	0.4808	0.5122	3

1. Denotes average heterozygosity estimated using nine microsatellite loci weighted by the ploidy level.

2. Denotes *Pokey* insertion site number estimated using TE display.

3. Denotes haploid *Pokey* number in 28S genes amplified using qPCR.

4. Denotes haploid *Pokey* number outside 28S genes amplified using qPCR.

5. Denotes *Pokey* insertion site number estimated using TE display minus the peak from elements in 28S genes.

6. Denotes total *Pokey* number outside 28S genes amplified using qPCR.

7. Denotes average heterozygosity of *Pokey* insertions outside 28S genes amplified using qPCR.

8. AF refers to Additional Files.

*P values are significant results.

### qPCR analysis of rRNA gene and *Pokey* copy number

Using the qPCR technique, we estimated the haploid number of 18S genes, 28S genes (Additional file 6), and *Pokey* inserted in 28S genes (r*Pokey*) and in the whole genome (t*Pokey*) in 19 isolates including 9 diploids and 10 polyploids (Table 2, Additional file 1). Using these estimates, we calculated the number of *Pokey* insertions outside 28S genes (g*Pokey* = t*Pokey* - r*Pokey*). Under the assumption that *Tif* or *Gtp* reference genes were neither duplicated or lost in any of the isolates, we expect the *Tif:Gtp* ratio to be close to 1, and this was the case with ratios ranging from 0.79 to 1.03 and a mean of 0.90 for diploids and 0.92 for polyploids (Table 2). It is unlikely that correlated losses or duplications of both genes would occur in multiple isolates and so we have assumed that diploids have two copies and triploids have three copies of each reference gene.

Both 18S and 28S genes showed a tendency towards a higher copy number per haploid genome in diploids than in polyploids (292.78 ±111.19 *vs*. 248.04 ±62.34 for 18S genes and 488.28 ±201.36 *vs*. 398.10 ±113.86 for 28S genes; Table 2), but differences between diploids and polyploids were not significant (Student’s *t*-test; *t* =1.0828, *df* = 17, *P* = 0.2940 for 18S and Student’s *t*-test; *t* =1.2337, *df* = 17, *P* = 0.2341 for 28S). The estimates of 18S and 28S number within each isolate were significantly correlated (Additional file 6) but the slopes of the lines generated by plotting them relative to one another were above the expected value of 1.0 with values of 1.80 for diploid hybrids and 1.66 for polyploids (Table 3). It is possible that we overestimated the number of 28S genes (Additional file 6) and so the number of 18S genes was used as a proxy of rDNA copy number in all subsequent analyses.

Excluding isolates with unknown hybrid nature, the average haploid number of t*Pokey* insertions was 17.81 ±4.35 for diploid hybrids and 15.58 ±3.43 for polyploid hybrids (Table 2) and the difference was not statistically significant (Student’s *t*-test; *t* = 1.1092, *df* = 13, *P* = 0.2947).

The number of r*Pokey* was higher in diploids than in polyploids (mean 5.19 *vs*. 3.10, respectively), but this difference was not significant (Student’s *t*-test; t = 1.0735, *df* = 13, *P* = 0.3026). Variation in the number of r*Pokey* insertions was higher in diploid than in polyploid hybrids (SD 5.03 *vs*. 1.07) but the difference was not statistically significant (Levene’s test, *W* = 2.0149, *P* = 0.1793). No correlation was found between the number of r*Pokey* and the number of 18S genes for either ploidy level (Table 3; Additional file 7) even if the outlier PX2-MB-1, which possesses a high number of r*Pokey* (16.5) and a low number of 18S genes compared to other diploids (Table 3), was omitted from the analysis.

The mean number of g*Pokey* was 12.63 in diploid hybrids and 12.48 in polyploid hybrids (Table 2) and the difference was not significant (Student’s *t*-test; *t* = 0.0653, *df* = 13, *P* = 0.9489). Variation in the number of g*Pokey* insertions was higher in diploid than in polyploid hybrids (SD 5.04 *vs*. 3.20) but the difference was not statistically significant (Levene’s test, *W* = 0.8830, *P* = 0.3645).

No significant correlation was found between the number of g*Pokey* and the number of r*Pokey* in hybrids of either ploidy level (Table 3; Additional file 8). The negative (but not significant) relationship between r*Pokey* and g*Pokey* in diploids was partly due to the high number of r*Pokey* in the isolate PX2-MB-1. However, the relationship was still negative and was nearly significant when this isolate was discarded from the analysis (Table 3; Additional file 8).

### Comparison of TE Display and qPCR

**Figure 3:**
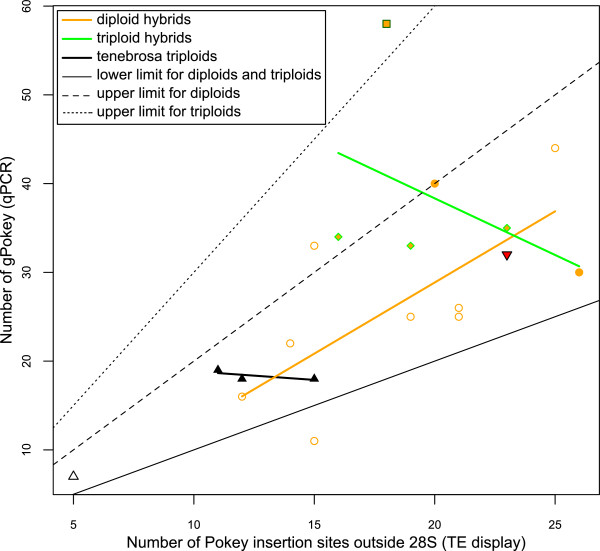
**Relationship between the total number of *Pokey *insertions outside rDNA estimated using qPCR and TE display.** Empty symbols are diploids and solid symbols are polyploids. Empty orange circles: diploid hybrids with *D. pulex* mitochondrial haplotypes; solid orange circles: triploid hybrids with *D. pulex* mitochondrial haplotypes; solid square: *D. middendorffiana*; empty black triangles: diploid *D. tenebrosa*; solid black triangles: triploid *D. tenebrosa*; solid red triangle: introgressed *D. tenebrosa* with a *D. pulex* nuclear genome; solid green diamond filled with orange: triploid hybrids with *D. pulicaria* mitochondrial haplotypes. Thin lines represent lower (slope = 1 and y-intercept = 0) and upper limits for diploids (slope = 2 and y-intercept = 0) and triploids (slope = 3 and y-intercept = 0) of *Pokey* insertion number (see Methods). Thick lines are linear regressions estimated from the data.

The assumption that the g*Pokey* elements amplified using qPCR and TE display are identical seems to be reasonable (Figure 3). Of all 19 isolates, only one (PX2-MI-7) had a ratio (k*n_*g*Pokey_)/n_TED-1_ below 1 which may indicate an overestimation of g*Pokey* number using TE display compared to qPCR. One diploid isolate (PX2-QC-2) had a ratio above 2 and a triploid isolate (MI3-MB-2) had a ratio above 3 (Figure 3). This suggests that either the number of g*Pokey* was overestimated by qPCR, underestimated by TE display, or both. The relationship between g*Pokey* number based on qPCR and TE display is positive and significant in diploid hybrids (Table 3; Figure 3). This relationship was negative but not significant in triploid *D. tenebrosa* and in triploid hybrids (Table 3; Figure 3).

After excluding isolates outside the lower and upper limits of possible values of total g*Pokey* insertions estimated with qPCR and TE display (values of the ratio (k*n_*gPokey*_)/*n*_*TED-1*_ between 1 and 2 for diploids and between 1 and 3 for triploids), the average heterozygosity across *Pokey* insertions loci among diploid hybrids is 59.85%. The relationship between the heterozygosity of *Pokey*-inserted loci and the number of g*Pokey* estimated using TE display is slightly negative but not significant (Table 3; Additional file 9). The slope of the relationship between the ploidy-weighted heterozygosity using nine microsatellite loci and the average heterozygosity of *Pokey*-inserted loci is negative and is significant for diploid hybrids (Table 3, Additional file 10).

## Discussion

### The polymorphism of *Pokey* insertion sites in *Daphnia* isolates

The polymorphism of TE insertion sites may depend on multiple factors such as selective pressure, drift, recombination rate, ploidy level, genomic background (*i.e.,* the parental origins of the hosts), geographic location, and the characteristics of the element(s) hosted in the genome [8,25,31,73,87-94]. If the diversity of *Pokey* insertion sites is due to the admixture of haploid genomes from different species with different architecture (that is nucleotide variation, number of repetitive genetic structures, etc.), the similarity of *Pokey* profiles is expected to mirror the genetic relationship of their hosts. According to the PCoA (Figure 1), the pairwise distance between *Pokey* profiles of the *Daphnia* isolates is not congruent with their pairwise genetic distance based on nine microsatellite loci if only the two first axes are taken into account. Similarities between the patterns produced by TE display and microsatellite analyses can only be revealed if the third axis of the PCoA is taken into account (Additional file 4). According to the K-means analysis, clusters based on similarity of *Pokey* profiles are not congruent with clusters based on microsatellite genotypes. Conversely, Mantel tests indicated that similarity between *Pokey* profiles is partially correlated with distance matrices constructed from microsatellite diversity and with mitochondrial haplotype diversity (*r* = 0.3957 and *r* = 0.3047, respectively).

The polymorphism of *Pokey* insertion sites in the isolates studied here imperfectly follows their evolutionary relationship with one another. This is concordant with previous results in which sequences from r*Pokey* elements amplified from some of the isolates included in this study show a different reticulation history than the one described by microsatellite data [71]. For example, r*Pokey* sequences from triploids PC3-QC-1 and PX3-QC-1, whose *Pokey* profiles cluster together using the K-mean analysis (Figure 1A), have *Pokey* sequences that are similar (Figure two in [71]). Similarly, r*Pokey* sequences from triploid isolates from Churchill (MI3-MB-2 and PC3-MB-5), whose *Pokey* profiles cluster with *D. tenebrosa* and diploid hybrid isolates in the K-means analysis (Figure 1A), are recombinant and show signatures of hybridization between *D. tenebrosa* and *D. pulex* or *D. pulicaria*. However, there was no indication of hybridization based on the analysis of microsatellite data, which clustered all *D. tenebrosa* isolates with one another (Figure 1B). Weider et al. [66] hypothesized introgression between *D. tenebrosa* and *D. pulex* or *D. pulicaria* based on mitochondrial DNA and allozyme analyses. The polymorphism of *Pokey* profiles may then mirror hybridization or introgression events between these species that microsatellites do not display due to homoplasy or null alleles in the microsatellite dataset. In our study, all *D. tenebrosa* isolates may be of hybrid origins but can still ordinate separately in the PCoA and cluster together in a separate group using the K-means analysis due to the sharing of a specific allele belonging to the *D. tenebrosa* species. Alternatively, *Pokey* insertion profiles may not correspond to the genetic relationships of their host due to genomic rearrangements and random loss of copies in the course of evolution.

Patterns of *Pokey* insertion site polymorphism cannot be explained solely by ploidy level as K-means analyses show that individuals with different ploidy levels group in the same cluster whereas triploid individuals may belong to different clusters (Figure 1A). Valizadeh and Crease [15] did not find a relationship between the similarity of *Pokey* profiles and the mode of reproduction using a Neighbor-Joining tree of *Pokey* profiles from cyclic and obligate isolates of *D. pulex*. They concluded that the absence of a relationship was due to the multiple origins of obligate parthenogenetic lineages from multiple cyclical parthenogenetic populations. Similarly, the absence of a relationship between similarity of *Pokey* profiles and ploidy level is likely due to the multiple and independent origins of polyploid isolates.

### Is *Pokey* load higher in *Daphnia* polyploids than diploids?

Our study examined the load of *Pokey* insertions in relation to ploidy level in natural populations of *Daphnia*. Both our diploid and polyploid isolates are hybrids (with the exception of some *D. tenebrosa* isolates that were excluded from the analyses of load) and are obligate parthenogens. These characteristics allow us to test the effect of ploidy level on the load of a class II transposable element without the confounding effects of hybridization *per se* and of different modes of reproduction. No significant differences in the density of *Pokey* insertions using either qPCR (per haploid genome) or TE display were found between diploid and polyploid hybrids, suggesting that an increase in ploidy level does not lead to an increase of *Pokey* insertions in the long term. The isolates studied were sampled from natural populations and the age of these clones is unknown. Previous studies have suggested that obligately parthenogenetic populations of *D. pulex* originated some 150,000 years ago [93] and that some polyploids from the *D. pulex* complex were produced during the Pleistocene [49]. It is possible that an increase in *Pokey* insertions occurs shortly after polyploidization as predicted by several hypotheses [25,28,94], but that genomic reorganization results in the loss of *Pokey* insertions with time. Loss of TE insertions following polyploidization (in both the short and long term) seems to be the rule rather the exception in most allopolyploid plants [26], regardless of whether the TEs are active. Loss of TE insertions is thought to be due to genome rearrangements via unequal and ectopic recombination events between TEs at non-homologous loci. Therefore, the non-significant difference between diploid and polyploid hybrids may be due to loss of *Pokey* following polyploidization. Conversely, the absence of statistical significance may be due to the substantial variability in the number of *Pokey* in both groups, which may be due to high variability of *Pokey* load in the parents. For example, Eagle and Crease [84] surveyed 69 non-hybrid isolates of *D. pulex* and *D. pulicaria* from 22 sampling sites and found that g*Pokey* number can vary from 4 to 24. Thus, g*Pokey* number in hybrids between these species will also vary according to the g*Pokey* load in their ascendants. Alternatively, the presence or absence of active *Pokey* elements in parental species may influence the subsequent proliferation of *Pokey* in the hybrid offspring and increase the variability of *Pokey* insertion sites in hybrids. However, if *Pokey* is not active during apomixis, it cannot proliferate in obligately parthenogenetic hybrid lineages – except perhaps through ameiotic recombination events.

It has been suggested that *Pokey* is not active in non-hybrid obligately parthenogenetic isolates but may be active in cyclically parthenogenetic isolates of *D. pulex*[15,70] and *D. pulicaria*[84]. Even so, it is possible that *Pokey* may be active in hybrids at least in the first generations after their formation due to the presence of active *Pokey* in their ascendants. Therefore, increases in the density of *Pokey* insertions may depend on the activity of *Pokey* and the effectiveness of regulation of *Pokey* in hybrid genomes [21,95-97]. Testing the activity of *Pokey* in diploid hybrids and performing additional studies on a larger number of hybrid and non-hybrid isolates will enhance our understanding of the dynamics and increase, if any, of *Pokey* elements in *D. pulex* × *D. pulicaria* hybrids.

There is no difference between the load of *Pokey* insertion sites based on TE display in the genomes of polyploid hybrids (20.60 ±3.47 at 50°C and 16.33 ±1.75 at 55°C) compared to diploid hybrids (18.09 ±3.99 at 50°C and 13.83 ±4.22 at 55°C). Similarly, there is no difference in the density of g*Pokey* per haploid genome between polyploid (12.48 ±3.20) and diploid (12.63 ±5.04) hybrids based on qPCR (Table 2). Conversely, the number of singletons (TE display at 50°C) is slightly higher in polyploids (20.6%) than in diploids (12.3%). The relationship between *Pokey* number and heterozygosity also differs between the two groups. g*Pokey* number (qPCR) increases with an increase in *Pokey* insertion sites (TE display) in diploids (Figure 3) but decreases in triploids (although not significantly). Moreover, in diploids, *Pokey* heterozygosity tends to decrease, though not significantly, as the number of different *Pokey* insertion sites increases (Table 3, Additional file 9). In contrast, polyploid heterozygosity increases (Table 3) and the number of total g*Pokey* insertions (qPCR) decreases when *Pokey* insertion sites (TE display) increases (Table 3, Figure 3). Taken together, these results may reflect relaxed selection on insertions at some sites in polyploid compared to diploid hybrids, as suggested by the genomic niche redundancy hypothesis [24,25]. For example, if two functioning copies of a gene are necessary and sufficient for survival of the host, a third copy could become a potential genomic niche for TEs in triploid individuals. If so, then no TEs should be inserted in this gene in diploids but one gene copy could carry an insert in triploids without causing a decrease in host fitness. Alternatively, the difference in the number of singleton sites between the two ploidy levels may simply be a consequence of insertion site polymorphism contributed by the additional chromosome sets carried by polyploids.

### *Pokey* in rDNA

The mean haploid number of 18S is higher (although not significant) in diploid hybrid isolates than in polyploid hybrids, but when the haploid rDNA copy number is multiplied by the ploidy level, the average is equal between diploid (586.5) and polyploid (586.5) hybrids with *D. pulex*, *D. pulicaria* or *D. middendorffiana* mtDNA haplotypes. Previous studies have shown that polyploid plant species, such as natural and artificial allotetraploid populations of *Tragopogon*[98] and allotetraploid and allohexaploid grass species [99], may experience rDNA rearrangement, including loss of rRNA gene copies. As most organisms have many more rRNA genes than they require for survival [100], it is possible that a loss of copies in polyploids is not harmful. Indeed, it is possible that selection could actually favor the loss of copies if polyploidization initially results in high rDNA copy numbers that are somewhat deleterious. It is noteworthy that the average haploid 18S number (293.3 with values from 131.5 to 451) in the diploid hybrid isolates is more than 30% higher than the average haploid 18S number in the non-hybrid diploid isolates of *D. pulex* (221.0 with values from 94 to 489.5) and *D. pulicaria* (217.3 with values from 97 to 444) studied by Eagle and Crease [84] using the same qPCR protocol. This difference is not statistically significant (one-way ANOVA; *F* = 2.418, *df* = 2, *P* = 0.0961). However, the number of *Daphnia* diploid hybrids tested here is low (n = 8) comparing to *D. pulicaria* (n = 37) and *D. pulex* (n = 43) isolates tested in the study by Eagle and Crease [84]. There is a high level of variation within each group and it will be interesting to confirm this pattern after analysis in a larger sample of diploid hybrids and laboratory-produced hybrids.

The number of 28S genes with r*Pokey* insertions accounts for an average of 1.93% of rDNA (with only one isolate, PX2-MB-1, above 5%; Table 2; Additional file 1), which is consistent with the results of Eagle and Crease [84] who found r*Pokey* insertions in approximately 1% of rDNA units in non-hybrid *D. pulex* and *D. pulicaria* isolates. Moreover, and still in accordance with Eagle and Crease [84], we did not find a correlation between the number of r*Pokey* and rDNA in *Daphnia* hybrid isolates, including the polyploids in which rDNA copy number per haploid genome is lower. This is consistent with the hypothesis that *Pokey* is not highly active in the rDNA of these species, and its number does not increase with the number of rDNA units. Even so, selection is not so efficient that it eliminates deleterious elements present at low copy number in a highly repetitive gene family [84,101,102].

### The effect of hybridization on ***Pokey*** load

Valizadeh and Crease [15] found a significantly lower (one-way ANOVA; *F* =67.65, *df* =3, *P* <0.001) average number of *Pokey* insertion sites in obligately and in cyclically parthenogenetic diploid isolates of *D. pulex* (3.27 ±2.07, n = 22 and 5.18 ±2.24, n = 22 respectively) compared to our survey of 12 obligately parthenogenetic hybrid isolates using the same annealing temperature (55°C) in TE display (13.83 ±4.22 for six diploid hybrid isolates and 16.33 ±1.75 for six polyploid hybrid isolates; Table 2). In addition, the qPCR estimate of g*Pokey* density is higher (although not statistically significant; one-way ANOVA; *F* = 2.549, *df* = 2, *P* = 0.085) in our reduced data set of diploid hybrid isolates (12.63 ±5.04 for eight diploid hybrids) than in the diploid non-hybrid isolates analyzed by Eagle and Crease (9.6 for *D. pulex*, n = 43, and 9.5 for *D. pulicaria*, n = 37) [84]. Therefore, there seems to be an increase in the density of *Pokey* insertions in the genomes of hybrid *Daphnia*. This increase could occur either during the early generations after hybridization by bursts of *Pokey* activity or, if *Pokey* elements are still active in hybrids, over a long period through the slow accumulation of *Pokey* insertions within the genome. Bursts of TE activity in hybrids have been highlighted in numerous homoploid hybrid plants [22,29], fruit flies [19,21,36] and wallabies [20]. In *Drosophila melanogaster* and *D. virilis*, hybrid dysgenic crosses may lead to bursts in activity of various TEs [21,97] due to release from cytoplasmic repression [37,95,96,103]. Interestingly, there is a slight trend of decreasing *Pokey* site heterozygosity (Table 3; Additional file 10) and a trend of increasing *Pokey* insertions sites (Figure 2) as host average heterozygosity increases. These trends suggest there may be increased activity of *Pokey* in hybrids that have the most evolutionarily divergent parents. Alternatively, increased genome and cell size favored by natural selection in new and/or stressful habitats may lead to a slow increase in the number of TEs in the genome [104]. Genetic drift may also lead to a slow increase in the number of TEs as suspected in sunflowers [104]. Three hybrid species of sunflower inhabiting harsh environments show genome size expansion due to proliferation of numerous class I TEs (retrotransposons) [34]. However, the proliferation of TEs is rare in contemporary natural sunflower hybrid populations and in artificial hybrid crosses [23,33], which suggest an increase of TEs after hybrid establishment via population processes such as genetic drift or natural selection. Selection in marginal habitats or drift following hybridization could also lead to an increase of TE density in hybrid *Daphnia* genomes.

## Conclusions

Using TE display and qPCR, we were able to describe insertion site polymorphism and the load of *Pokey* elements in diploid and polyploid hybrid isolates of the *D. pulex* species complex. The polymorphism of *Pokey* insertion sites was not congruent with the evolutionary history and genetic relationships of their hosts. Diploid and polyploid hybrids did not differ significantly in the number of *Pokey* insertions, using either qPCR or TE display, as has been shown in studies comparing diploid and polyploid plants. The number of singletons estimated with TE display is slightly higher in polyploid than in diploid hybrids. Together, these results may reflect a higher number of sites available for *Pokey* insertions in polyploid than in diploid hybrids, or an increase in polymorphism due to the combination of genomes with *Pokey* at different insertion sites. Compared to previous studies on *Pokey* in the *D. pulex* complex, we found the density of *Pokey* insertions per haploid genome to be higher in obligately parthenogenetic hybrids (both diploids and polyploids) than in non-hybrid diploids (either cyclical or obligate parthenogens) leading to the conclusion that hybridization may lead to an overall increase in *Pokey* insertions. The estimation of polymorphism and TE load in laboratory-produced hybrids and the analysis of additional samples of hybrids will provide more insight into the population dynamics of TEs in diploid and polyploid hybrids of *Daphnia*.

## Abbreviations

D. pulex: *Daphnia pulex*; gPokey: Genomic *Pokey* elements inserted outside rDNA; Ldh: Lactate dehydrogenase gene; PCoA: Principal coordinate analysis; qPCR: Quantitative PCR; rPokey: *Pokey* elements inserted in rDNA; rDNA: ribosomal DNA; TE: Transposable element; TED: Transposable element display.

## Competing interests

The authors declare that they have no competing interests.

## Authors’ contributions

RV designed the project and wrote the manuscript. RV and SHCE planned the analyses. RV and SHCE conducted the analyses. All authors analyzed the data and contributed to the writing and editing of the manuscript. All authors approved the final manuscript.

## Supplementary Material

Additional file 1 **Characteristics of the *Daphnia *** **isolates used in this study.** Labels of the isolates are composites of their characteristics. The first two letters represent the mitochondrial haplotype followed by the ploidy level (2× or 3×), a 2-letter country or state/province code and the isolate number. Mitochondrial haplotypes are as follows: EPC = Eastern *D. pulicaria*, WPC = Western *D. pulicaria*, PPC = Polar *D. pulicaria*, PanPX = Panarctic *D. pulex*, MIDD = *D. middendorffiana sensu stricto*, TENE = *D. tenebrosa*). Ldh is the Lactate dehydrogenase genotype and indicates the hybrid nature of each isolate. H_pl_ is the ploidy-weighted heterozygosity. rRNA gene and *Pokey* number were determined using TE display and qPCR; 50°C and 55°C are the annealing temperatures used to generate the PCR amplicons in TE display. Total *Pokey* = all *Pokey* elements in the genome. rDNA *Pokey* = *Pokey* elements in 28S rRNA genes. Genomic *Pokey* = total -rDNA elements. TG ratio is the number of *Tif* relative to the number of *Gtp* single copy reference genes. Click here for file

Additional file 2 Supplementary material and methods describing TE display and qPCR protocols. Click here for file

Additional file 3 **TE display profiles of diploid and polyploid isolates in the *D. pulex *** **species complex.** Sum of the amplification signals (peaks) are presented for each isolate. Click here for file

Additional file 4 **Three-dimensional representation of Principal Coordinate Analyses of Jaccard distance matrix of *Pokey *** **profiles and Bruvo distance matrix of microsatellite diversity in diploid and polyploid isolates of the *Daphnia pulex *** **complex.** (A) *Pokey* profiles generated using TE display; (B) Microsatellite genotypes determined by Vergilino et al. [45]. The three first axes are represented. Empty symbols are diploids and solid symbols are polyploids. Empty orange circles: diploid hybrids with pulex mitochondrial haplotype, solid orange circles: triploid hybrids with *D. pulex* mitochondrial haplotype, solid square: *D. middendorffiana sensu stricto*, empty black triangles: diploid *D. tenebrosa*, solid black triangles: triploid *D. tenebrosa*, solid red triangle: introgressed *D. tenebrosa* with *D. pulex* nuclear genome, solid green diamond filled with orange: triploid hybrids with *D. pulicaria* mitochondrial haplotype; (C) and (D) are screeplots and represent the eigenvalues of the axes of Principal Coordinate Analysis (A) and (B), respectively. Click here for file

Additional file 5 **Results of the covariance analysis (ANCOVA) of TE display results from diploid and polyploid isolates in the *Daphnia pulex *** **complex.** Ploidy level and ploidy-weighted heterozygosity (*H*_*pl*_) were used as the independent variables. Click here for file

Additional file 6 **Relationship between 18S and 28S gene number in diploid and polyploid isolates of the *Daphnia pulex *** **complex.** (A) Histograms of haploid number of 18S and 28S genes in each isolate. (B) Correlation between 18S and 28S gene number. Symbols represent mitochondrial haplotypes: circles for isolates with *D. pulex* mitochondria, diamonds for isolates with *D. pulicaria* mitochondria, squares for isolates with *D. middendorffiana* mitochondria, triangles for isolates with *D. tenebrosa* mitochondria and inverted triangles for introgressed *D. tenebrosa*. Empty symbols represent putative diploids and solid ones indicate polyploids. Dashed and solid lines are linear regressions estimated from the data in diploids and polyploids, respectively. The dotted line was generated by plotting 18S gene number on both axes. Click here for file

Additional file 7 **Correlation between haploid number of *Pokey* in rDNA (r*Pokey*) and haploid 18S gene number in diploid and triploid isolates of the *D. pulex *** **species complex.** Empty circles represent diploid isolates and solid circles represent triploid isolates. Red empty circle represents the diploid isolate PX2-MB-1. Dashed and solid lines are linear regressions estimated from the data. The dashed lines with long strokes represent the linear regression following diploid hybrids without the isolate PX2-MB-1. Click here for file

Additional file 8 **Correlation between the haploid number of *Pokey *** **in rDNA (r*Pokey*) and the haploid number of *Pokey *** **in other genomic locations (g*Pokey*) in diploid and triploid isolates of the *D. pulex *** **species complex.** Empty circles represent diploid isolates and solid circles represent triploid isolates. Red empty circle represents the diploid isolate PX2-MB-1. Dashed and solid lines are linear regressions estimated from the data. The dashed lines with long strokes represent the linear regression following diploid hybrids without the isolate PX2-MB-1. Click here for file

Additional file 9 **Correlation between average *Pokey *** **insertion site heterozygosity (*H***_***gPokey***_**) and the number of *Pokey *** **insertions outside rDNA based on TE display analysis of diploid isolates.** The dashed line represents the linear regression estimated from the data. Click here for file

Additional file 10 **Correlation between average *Pokey *** **insertion site heterozygosity (*H***_***g*Pokey**_**) and heterozygosity (*H***_***pl***_**) of their diploid hosts based on microsatellite loci.** The dashed line represents the linear regression estimated from the data. Click here for file
