# Supplementary material for: Impact of ploidy level on the distribution of Pokey element insertions in the Daphnia pulex complex
Source: Mob DNA. 2014 Jan 2;5:1. doi: 10.1186/1759-8753-5-1 (PMC3882798; doi:10.1186/1759-8753-5-1)
Supplement: Additional file 1 — Characteristics of the Daphnia isolates used in this study. Labels of the isolates are composites of their characteristics. The first two letters represent the mitochondrial haplotype followed by the ploidy level (2× or 3×), a 2-letter country or state/province code and the isolate number. Mitochondrial haplotypes are as follows: EPC = Eastern D. pulicaria, WPC = Western D. pulicaria, PPC = Polar D. pulicaria, PanPX = Panarctic D. pulex, MIDD = D. middendorffiana sensu stricto, TENE = D. tenebrosa). Ldh is the Lactate dehydrogenase genotype and indicates the hybrid nature of each isolate. Hpl is the ploidy-weighted heterozygosity. rRNA gene and Pokey number were determined using TE display and qPCR; 50°C and 55°C are the annealing temperatures used to generate the PCR amplicons in TE display. Total Pokey = all Pokey elements in the genome. rDNA Pokey = Pokey elements in 28S rRNA genes. Genomic Pokey = total -rDNA elements. TG ratio is the number of Tif relative to the number of Gtp single copy reference genes. [file 1759-8753-5-1-S1.pdf]

Additional file 1. Characteristics of the *Daphnia* isolates used in this study. Labels of the isolates are composites of their characteristics. The first two letters represent the mitochondrial haplotype followed by the ploidy level (2x or 3x), a 2-letter country or state/province code and the isolate number. Mitochondrial haplotypes are as follows: EPC = Eastern *D. pulicaria*, WPC = Western *D. pulicaria*, PPC = Polar *D. pulicaria*, PanPX = Panarctic *D. pulex*, MIDD = *D. middendorffiana sensu stricto*, TENE = *D. tenebrosa*). *Ldh* is the *Lactate dehydrogenase* genotype and indicates the hybrid nature of each isolate. H_{pl} is the ploidy-weighted heterozygosity. rRNA gene and *Pokey* number were determined using TE display and qPCR; 50°C and 55°C are the annealing temperatures used to generate the PCR amplicons in TE display. Total *Pokey* = all *Pokey* elements in the genome. rDNA *Pokey* = *Pokey* elements in 28S rRNA genes. Genomic *Pokey* = total -rDNA elements. TG ratio is the number of *Tif* relative to the number of *Gtp* single copy reference genes.

Isolate	Geographic location	Mitochondrial haplotype [ND5 accession number]	*Ldh*	Ploidy level	H_{pl}	TE display		qPCR					
						50°C	55°C	18S rDNA copy	28S rDNA copy	Total *Pokey*	rDNA *Pokey*	Genomic Pokey	TG ratio
PX2-MB-1	Churchill, Manitoba, CAN	PanPX [HQ434631]	SF	2x	0.39	13	6	131.5	218.5	24.5	16.5	8	0.88
PX2-ON-2	Windsor, Ontario, CAN	PanPX [HQ434649]	SF	2x	0.33	22		238.5	371	14.5	1.5	13.0	0.90
PX2-ON-3	Windsor, Ontario, CAN	PanPX [HQ434633]	SF	2x	0.39	14							
PX2-ON-4	Windsor, Ontario, CAN	PanPX [HQ434634]	SF	2x	0.28	18							
PX2-QC-5	Ste-Foy, Québec, CAN	PanPX [HQ434650]	SF	2x	0.39	20	16	382	658	15.5	3	12.5	0.98
PX2-QC-6	Ste-Foy, Québec, CAN	PanPX [HQ434651]	SF	2x	0.39	22	16	371.5	623	15	2.5	12.5	0.94

PX2-MI-7	Michigan, U.S.A.	PanPX [HQ434655]	SF	2x	0.44	16	14	451	787	13.5	8	5.5	0.87
PX2-QC-8	Kuujjarapik, Québec, CAN	PanPX [FJ591098]	SF	2x	0.39	15		354	575	15.5	4.5	11	0.97
PX2-QC-9	Métis, Québec, CAN	PanPX [HQ434663]	SF	2x	0.33	16	13	192.5	292	20.5	4	16.5	0.9
PX2-QC-10	Kuujjarapik, Québec, CAN	PanPX [FJ591109]	SF	2x	0.44	17							
PX2-QC-11	Kuujjarapik, Québec, CAN	PanPX [FJ591096]	SF	2x	0.50	26	18	225	365.5	23.5	1.5	22	0.79
PX3-QC-1	Kuujjarapik, Québec, CAN	PanPX [FJ591097]	SF	3x	0.33	21	16	178	285.34	17.34	4	13.34	0.83
PX3-QC-2	Kuujjarapik, Québec, CAN	PanPX [FJ591101]	SF	3x	0.50	27	19	153.34	213	11.34	1.34	10	0.96
PC3-QC-1	Kuujjarapik, Québec, CAN	EPC [FJ591110]	SF	3x	0.39	20							
PC3-QC-2	Kuujjarapik, Québec, CAN	EPC [FJ591111]	SF	3x	0.61	24		256.34	398	16.34	4.67	11.67	1.03
PC3-QC-3	Kuujjarapik, Québec, CAN	EPC [FJ591113]	SF	3x	0.67	16							
PC3-MB-4	Churchill, Manitoba, CAN	PPC [HQ434630]	SF	3x	0.33	20	17	178	281	13.67	2.67	11	0.98
PC3-MB-5	Churchill, Manitoba, CAN	PPC [FJ591102]	SF	3x	0.28	18							
PC3-MB-6	Churchill, Manitoba, CAN	WPC [FJ591106]	SF	3x	0.44	17	15	230.67	430	14.34	3	11.34	0.94
MI3-MB-2	Churchill, Manitoba, CAN	MIDD [FJ591122]	SF	3x	0.39	19	14	176	278.67	22	2.67	19.34	0.87
TE2-MB-1	Churchill, Manitoba, CAN	TENE [FJ591118]	/	2x	0.28	16							
TE2-MB-2	Churchill, Manitoba, CAN	TENE [FJ591123]	/	2x	0.22	12							
TE2-MB-3	Churchill, Manitoba, CAN	TENE [HQ434637]	/	2x	0.28	6	6	289	504.5	7.5	4	3.5	0.97
TE3-MB-1	Churchill, Manitoba, CAN	TENE [FJ591117]	/	3x	0.44	13	11	357	576	11.34	5.34	6	0.92

TE3-MB-2	Churchill, Manitoba, CAN	TENE [FJ591116]	/	3x	0.38	12	11	—	319.34	517.34	12	5.67	6.34	0.95
TE3-MB-3	Churchill, Manitoba, CAN	TENE [FJ591121]	/	3x	0.56	16	10		301.34	461	11	5	6	0.86
TE3-MB-4	Churchill, Manitoba, CAN	TENE [FJ591115]	SS	3x	0.67	24	17		330.34	540.67	14	3.34	10.67	0.83
