# Supplementary material for: Impact of ploidy level on the distribution of Pokey element insertions in the Daphnia pulex complex
Source: Mob DNA. 2014 Jan 2;5:1. doi: 10.1186/1759-8753-5-1 (PMC3882798; doi:10.1186/1759-8753-5-1)
Supplement: Additional file 2 — Supplementary material and methods describing TE display and qPCR protocols. [file 1759-8753-5-1-S2.pdf]

Additional File 2. Supplementary material and methods

TE Display

After genomic DNA extraction, we digested approximately 100 ng of genomic DNA with 5 units of *Bfa*I restriction enzyme (New England Biolabs, Ipswich, MA) in a total volume of 50 µL for 5 h at 37°C in 1× reaction buffer supplied by the manufacturer. The digested DNA was ligated to 25 pmol of forward and reverse *Bfa*I linkers (*Bfa*I linkerF 5′-TACTCAGGACTCAT and *Bfa*I linkerR 5′-GACGATGAGTCCTGAG) in a 20 µL reaction containing 400 units of T4 DNA Ligase (New England Biolabs, Ipswich, MA) in 1× T4 DNA Ligase buffer. The ligation reaction was incubated overnight at 4°C. The ligated DNA was amplified in a primary (pre-selective) PCR reaction of 50 µL total volume that contained 1 µL of the digested-ligated DNA sample, 1 pmol of the primer Pok6456F (5′-GACAACGGTGGCCGAAACGCGG, located near the 3′ end of the *Pokey* element), 1 pmol of the primer *Bfa*IR (5′-GACGATGAGTCCTGAGTAG), 2 mM $MgCl_2$, 1× PCR buffer (10 mM TrisHCl, pH 8.3, 50 mM KCl), 0.8 mM dNTPs, and 0.5 unit of Taq DNA polymerase (New England Biolabs). PCR reactions were performed in a PTC-100 Thermocycler (MJ Research, Waltham, MA) for 35 cycles each consisting of 30 sec of denaturing at 94°C, 90 sec of annealing at 50°C, and 1 min of extension at 72°C, with a final extension of 5 min at 72°C. Approximately 60-70 ng of the primary PCR product was used as the template for a secondary (selective) PCR amplification. This reaction was carried out under the same reaction conditions as above except that the total volume was 20 µL, only 0.25 unit of *Taq* DNA polymerase (New England Biolabs) was used,

and the forward primer was Pok6464F (5′-TGGCCAAAACACGGTTTGGCCG) labeled with the fluorescent dye, HEX (Applied Biosystems, Foster City, CA). This primer is just downstream of Pok6456F, at the 3′ end of the *Pokey* element. The secondary PCR products were resolved on an ABI 3730 DNA Analyzer (Applied Biosystems, Foster City, CA) in the Genomics Facility at the University of Guelph using . The Peak Scanner™ software version 1.0 was used to determine the number and the size of the peaks in each electropherogram. We only included fragments ≥160 bp as the minimum fragment size produced by the amplification of *Pokey* elements is 160 bp if there is a *Bfa*I restriction site immediately downstream of the TTAA *Pokey* insertion site [1]. To ensure that the TE display patterns were reproducible, we repeated each individual primary PCR reaction three times for each isolate. A separate secondary PCR reaction was performed from each of the triplicate primary PCR reactions, and the triplicate assays for each sample were compared to each other. We only included fragments with a peak height of 100 fluorescence units in subsequent analyses. Only signals that are present in at least two of the triplicate primary PCR reactions were included in the analysis.

qPCR

qPCR was used to estimate the haploid number of *Pokey* elements in the 28S rRNA genes, in the entire genome, and to calculate the haploid number of *Pokey* elements outside the 28S rRNA genes. This was done by comparing the rate of amplification of *Pokey* in the 28S rRNA genes and *Pokey* in the entire genome to

the rate of amplification of two single-copy genes, using four primer pairs (See Table 1). We determined the percent amplification efficiency (PAE) for each pair of primers using the standard curve method as described in Eagle and Crease [2] and Yuan et al. [3]. PAE is the percent amplification efficiency and is used to account for differences in primer pair amplification efficiency [3]. Reactions had a final volume of 20 µL, containing 1X Power SYBR® Green PCR Master Mix (Applied Biosystems), 0.25 pmol of each primer, and approximately 10 ng of genomic DNA and were run in triplicates for each gene for each isolate. The reactions were run on the StepOnePlus™ Real-Time PCR System (Applied Biosystems) starting with a 10 min initial denaturation at 95°C followed by 40 cycles of 95°C for 15 sec and 60°C for 1 min. The dissociation curve was generated by cooling to 60°C then heating to 95°C in 0.3°C increments.

All reactions were run in triplicate. If the triplicate variance exceeded 0.2, the value furthest from the mean was excluded from further analysis. The threshold amount of fluorescence was set to 0.2 for amplicons of 50 bp and adjusted for longer amplicons according the formula 2^[1-(50/length of amplicon in bp)] x 0.2 [2]. The cycle at which the amplification curve crosses the threshold is the cycle threshold, C_T, value. C_T values were examined in the StepOne v2.0 software (Applied Biosystems). To estimate the number of *Pokey* elements each multicopy gene C_T value (2 or 3) was compared to each single-copy gene C_T value (4 to 6) using the formula, $2^{\Delta CT}$ where ΔC_T is ((C_T x PAE $_{\textit{Pokey}\ \text{gene}}$)-($C_T$ x PAE $_{\text{single-copy gene}}$)), producing up to 18 estimates of gene number for each isolate. Means, rounded to the nearest 0.5 in diploids and 0.34 in triploids, and standard

deviations were calculated for each gene for each isolate using these 18 estimates. The number of *Pokey* outside the 28S rRNA genes was calculated as the number of *Pokey* in the entire genome – the number of *Pokey* in the 28S rRNA genes.

References

1. Valizadeh P, Crease TJ: **The association between breeding system and transposable element dynamics in *Daphnia pulex*.** *J Mol Evol* 2008, **66:**643-654.
2. Eagle SHC, Crease TJ: **Copy number variation of ribosomal DNA and *Pokey* transposons in natural populations of *Daphnia*.** *Mobile DNA* 2012, **3:**4.
3. Yuan JS, Wang D, Stewart CN: **Statistical methods for efficiency adjusted real-time PCR quantification.** *Biotech J* 2008, **3:**112-123.
