# Supplementary material for: Impact of ploidy level on the distribution of Pokey element insertions in the Daphnia pulex complex
Source: Mob DNA. 2014 Jan 2;5:1. doi: 10.1186/1759-8753-5-1 (PMC3882798; doi:10.1186/1759-8753-5-1)

Additional File 4

Three-dimensional representation of Principal Coordinate Analyses of Jaccard distance matrix of *Pokey* profiles and Bruvo distance matrix of microsatellite diversity in diploid and polyploid isolates of the *Daphnia pulex* complex. (**A**) *Pokey* profiles generated using TE display; (**B**) Microsatellite genotypes determined by Vergilino et al. [45]. The three first axes are represented. Empty symbols are diploids and solid symbols are polyploids. Empty orange circles: diploid hybrids with pulex mitochondrial haplotype, solid orange circles: triploid hybrids with *D. pulex* mitochondrial haplotype, solid square: *D. middendorffiana sensu stricto*, empty black triangles: diploid *D. tenebrosa*, solid black triangles: triploid *D. tenebrosa*, solid red triangle: introgressed *D. tenebrosa* with *D. pulex* nuclear genome, solid green diamond filled with orange: triploid hybrids with *D. pulicaria* mitochondrial haplotype; (**C**) and (**D**) are screenplots and represent the eigenvalues of the axes of Principal Coordinate Analysis (**A**) and (**B**), respectively.

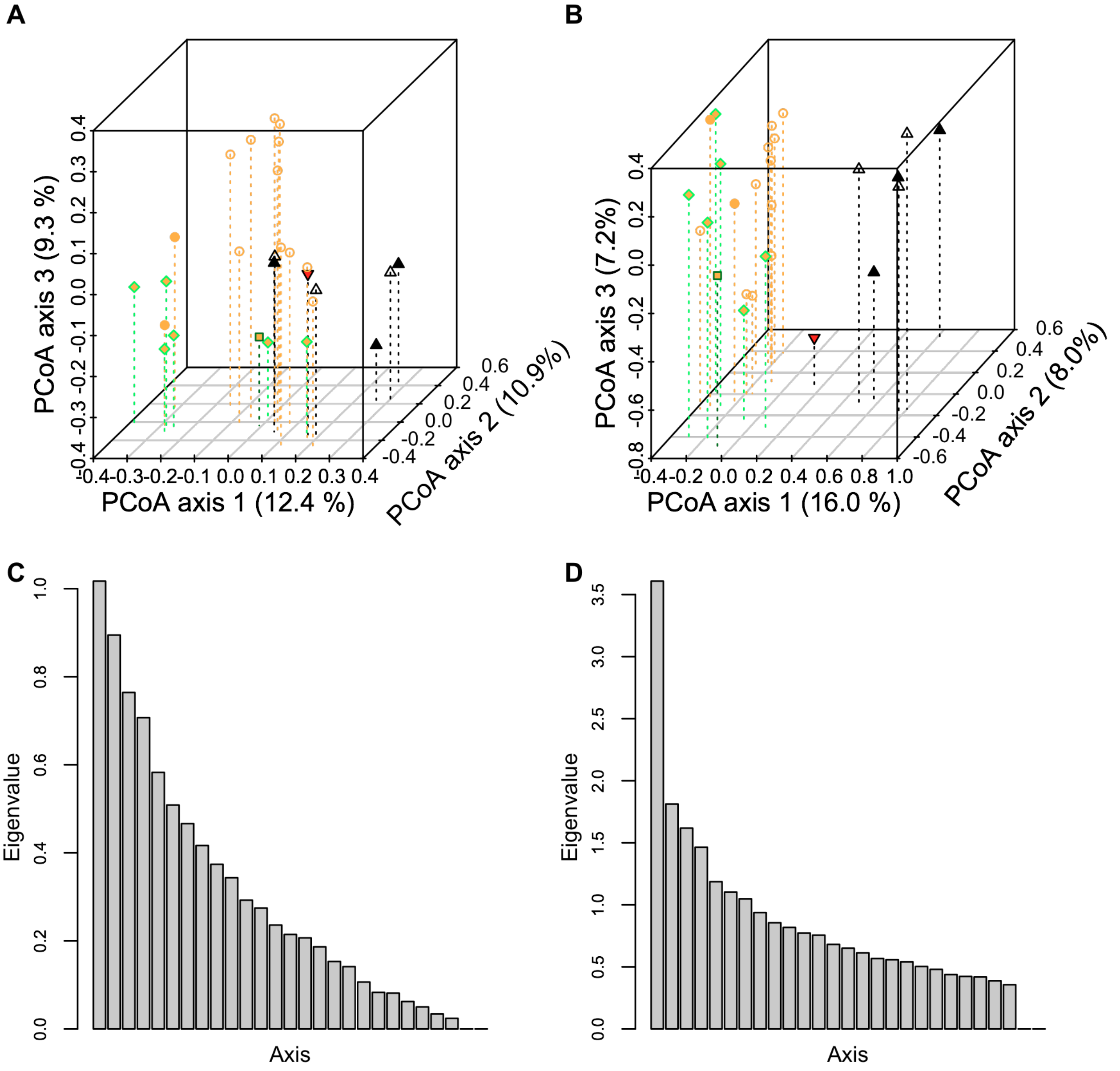

Supplement: Additional file 4 — Three-dimensional representation of Principal Coordinate Analyses of Jaccard distance matrix of Pokey profiles and Bruvo distance matrix of microsatellite diversity in diploid and polyploid isolates of the Daphnia pulex complex. (A) Pokey profiles generated using TE display; (B) Microsatellite genotypes determined by Vergilino et al. [45]. The three first axes are represented. Empty symbols are diploids and solid symbols are polyploids. Empty orange circles: diploid hybrids with pulex mitochondrial haplotype, solid orange circles: triploid hybrids with D. pulex mitochondrial haplotype, solid square: D. middendorffiana sensu stricto, empty black triangles: diploid D. tenebrosa, solid black triangles: triploid D. tenebrosa, solid red triangle: introgressed D. tenebrosa with D. pulex nuclear genome, solid green diamond filled with orange: triploid hybrids with D. pulicaria mitochondrial haplotype; (C) and (D) are screeplots and represent the eigenvalues of the axes of Principal Coordinate Analysis (A) and (B), respectively. [file 1759-8753-5-1-S4.pdf]
