# Supplementary material for: Impact of ploidy level on the distribution of Pokey element insertions in the Daphnia pulex complex
Source: Mob DNA. 2014 Jan 2;5:1. doi: 10.1186/1759-8753-5-1 (PMC3882798; doi:10.1186/1759-8753-5-1)
Supplement: Additional file 5 — Results of the covariance analysis (ANCOVA) of TE display results from diploid and polyploid isolates in the Daphnia pulex complex. Ploidy level and ploidy-weighted heterozygosity (Hpl) were used as the independent variables. [file 1759-8753-5-1-S5.pdf]

Additional File 5

Results of the covariance analysis (ANCOVA) of TE display results from isolates in the *Daphnia pulex* complex. Ploidy level and ploidy-weighted heterozygosity (H_{pl}) were used as the independent variables.

	df	Sum of Square	Mean square	F-value	Pr(>|F|)
H_{pl}	1	31.24	31.24	2.132	0.162
Ploidy	1	17.03	17.03	1.162	0.296
H_{pl} : Ploidy	1	3.00	3.00	0.205	0.657
Residuals	17	249.03	14.65	-	-
