# Supplementary material for: Impact of ploidy level on the distribution of Pokey element insertions in the Daphnia pulex complex
Source: Mob DNA. 2014 Jan 2;5:1. doi: 10.1186/1759-8753-5-1 (PMC3882798; doi:10.1186/1759-8753-5-1)
Supplement: Additional file 6 — Relationship between 18S and 28S gene number in diploid and polyploid isolates of the Daphnia pulex complex. (A) Histograms of haploid number of 18S and 28S genes in each isolate. (B) Correlation between 18S and 28S gene number. Symbols represent mitochondrial haplotypes: circles for isolates with D. pulex mitochondria, diamonds for isolates with D. pulicaria mitochondria, squares for isolates with D. middendorffiana mitochondria, triangles for isolates with D. tenebrosa mitochondria and inverted triangles for introgressed D. tenebrosa. Empty symbols represent putative diploids and solid ones indicate polyploids. Dashed and solid lines are linear regressions estimated from the data in diploids and polyploids, respectively. The dotted line was generated by plotting 18S gene number on both axes. [file 1759-8753-5-1-S6.pdf]

Additional File 6

Relationship between 18S and 28S gene number in diploid and polyploid isolates of the *Daphnia pulex* complex. (**A**) Histograms of haploid number of 18S and 28S genes in each isolate. (**B**) Correlation between 18S and 28S gene number. Symbols represent mitochondrial haplotypes: circles for isolates with *D. pulex* mitochondria, diamonds for isolates with *D. pulicaria* mitochondria, squares for isolates with *D. middendorffiana* mitochondria, triangles for isolates with *D. tenebrosa* mitochondria and inverted triangles for introgressed *D. tenebrosa.* Empty symbols represent putative diploids and solid ones indicate polyploids. Dashed and solid lines are linear regressions estimated from the data in diploids and polyploids, respectively. The dotted line was generated by plotting 18S gene number on both axes.

18S and 28S numbers were significantly different with 28S exceeding 18S in all 19 isolates tested (paired Student *t*-test, t=7.8006, df=13, p-value<0.0001, Additional File 5A). The intensity of SYBR® green dye signal is dependent on the length of the amplicons in qPCR analyses. The longer the amplicon, the more SYBR® green dye binds to it resulting in a brighter signal [83]. The amplicon size for quantifying 28S gene number (150 bp) is 3 times higher than the amplicon size of *Tif*, *Gtp* and the 18S gene (50 bp for each; Table 1). Thus, it is possible that we overestimated the number of 28S genes even though we attempted to compensate for the longer amplicon by adjusting the threshold at which the C_T value was determined [80].

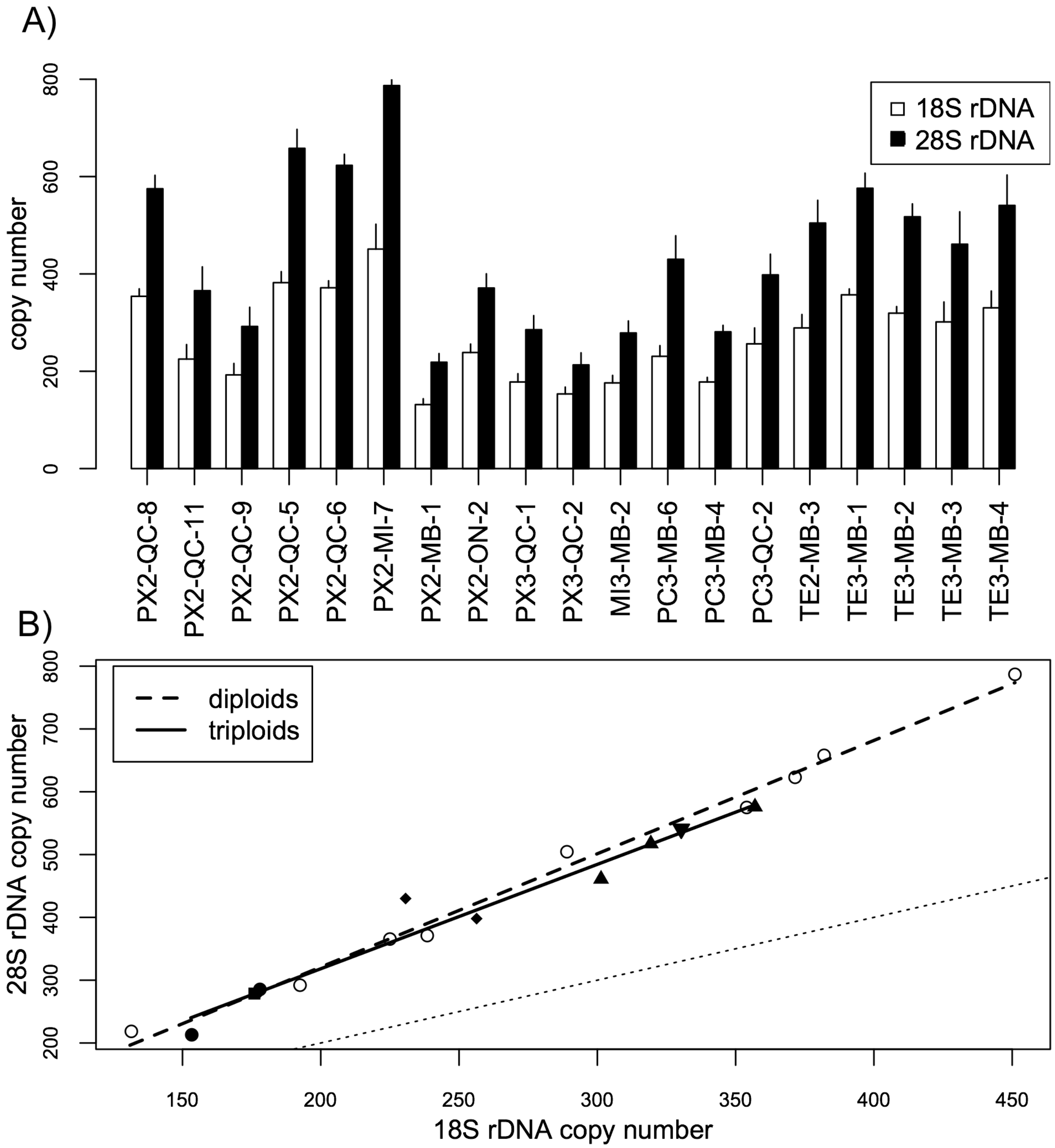
A)
copy number
0
200
400
600
800
18S rDNA
28S rDNA
PX2-QC-8
PX2-QC-11
PX2-QC-9
PX2-QC-5
PX2-QC-6
PX2-MI-7
PX2-MB-1
PX2-ON-2
PX3-QC-1
PX3-QC-2
MI3-MB-2
PC3-MB-6
PC3-MB-4
PC3-QC-2
TE2-MB-3
TE3-MB-1
TE3-MB-2
TE3-MB-3
TE3-MB-4
B)
28S rDNA copy number
200
300
400
500
600
700
800
diploids
triploids
150
200
250
300
350
400
450
18S rDNA copy number
