# Supplementary material for: Impact of ploidy level on the distribution of Pokey element insertions in the Daphnia pulex complex
Source: Mob DNA. 2014 Jan 2;5:1. doi: 10.1186/1759-8753-5-1 (PMC3882798; doi:10.1186/1759-8753-5-1)

Additional file 8

Correlation between the haploid number of *Pokey* in rDNA (r*Pokey*) and the haploid number of *Pokey* in other genomic locations (g*Pokey*) in diploid and triploid isolates of the *D. pulex* species complex. Empty circles represent diploid isolates and solid circles represent triploid isolates. Red empty circle represents the diploid isolate PX2-MB-1. Dashed and solid lines are linear regressions estimated from the data. The dashed lines with long strokes represent the linear regression following diploid hybrids without the isolate PX2-MB-1.

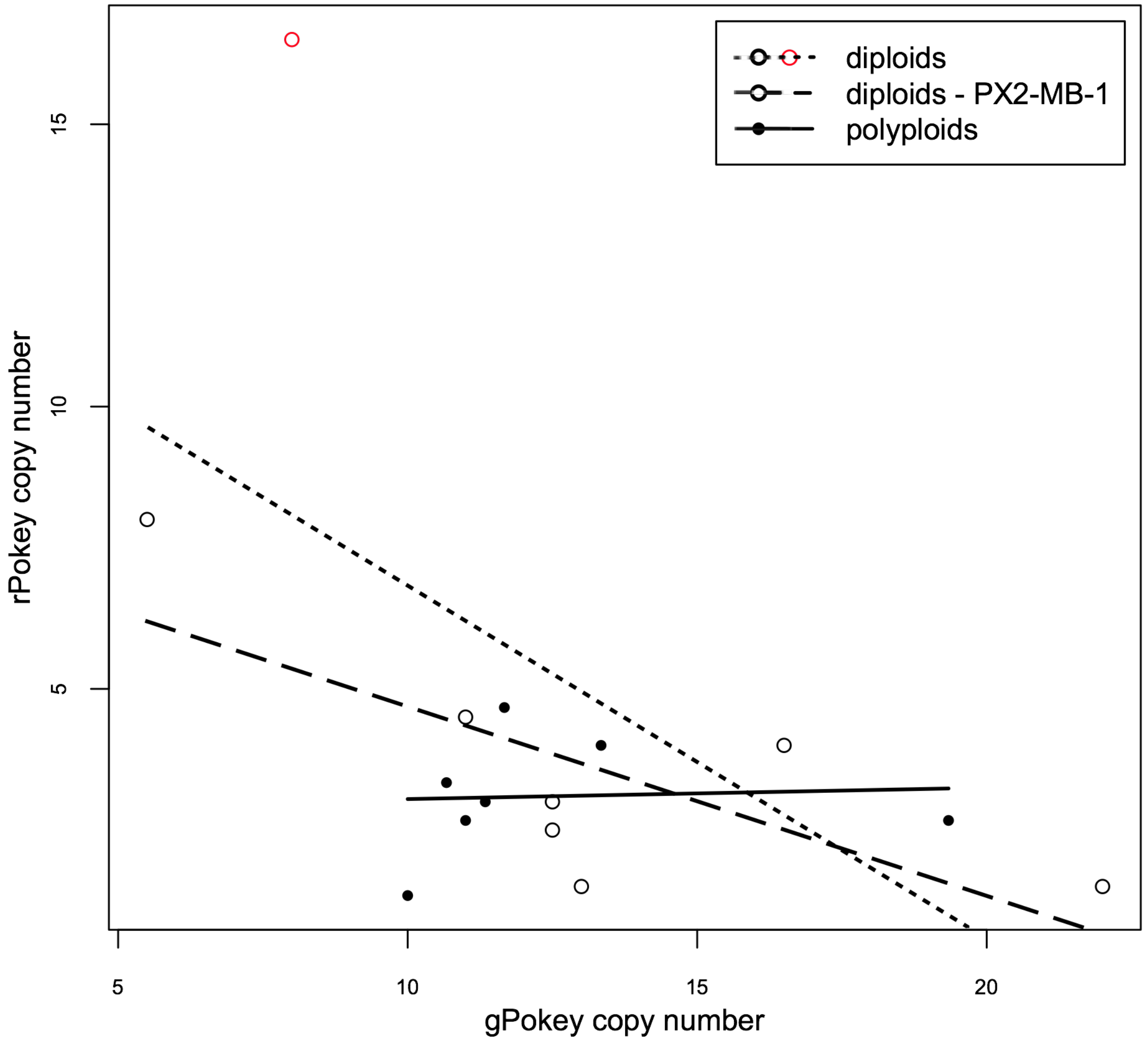

Supplement: Additional file 8 — Correlation between the haploid number of Pokey in rDNA (rPokey) and the haploid number of Pokey in other genomic locations (gPokey) in diploid and triploid isolates of the D. pulex species complex. Empty circles represent diploid isolates and solid circles represent triploid isolates. Red empty circle represents the diploid isolate PX2-MB-1. Dashed and solid lines are linear regressions estimated from the data. The dashed lines with long strokes represent the linear regression following diploid hybrids without the isolate PX2-MB-1. [file 1759-8753-5-1-S8.pdf]
