# Supplementary material for: Impact of ploidy level on the distribution of Pokey element insertions in the Daphnia pulex complex
Source: Mob DNA. 2014 Jan 2;5:1. doi: 10.1186/1759-8753-5-1 (PMC3882798; doi:10.1186/1759-8753-5-1)

Additional file 9

Correlation between average *Pokey* insertion site heterozygosity (H_{gPokey}) and the number of *Pokey* insertions outside rDNA based on TE display analysis of diploid isolates. The dashed line represents the linear regression estimated from the data.

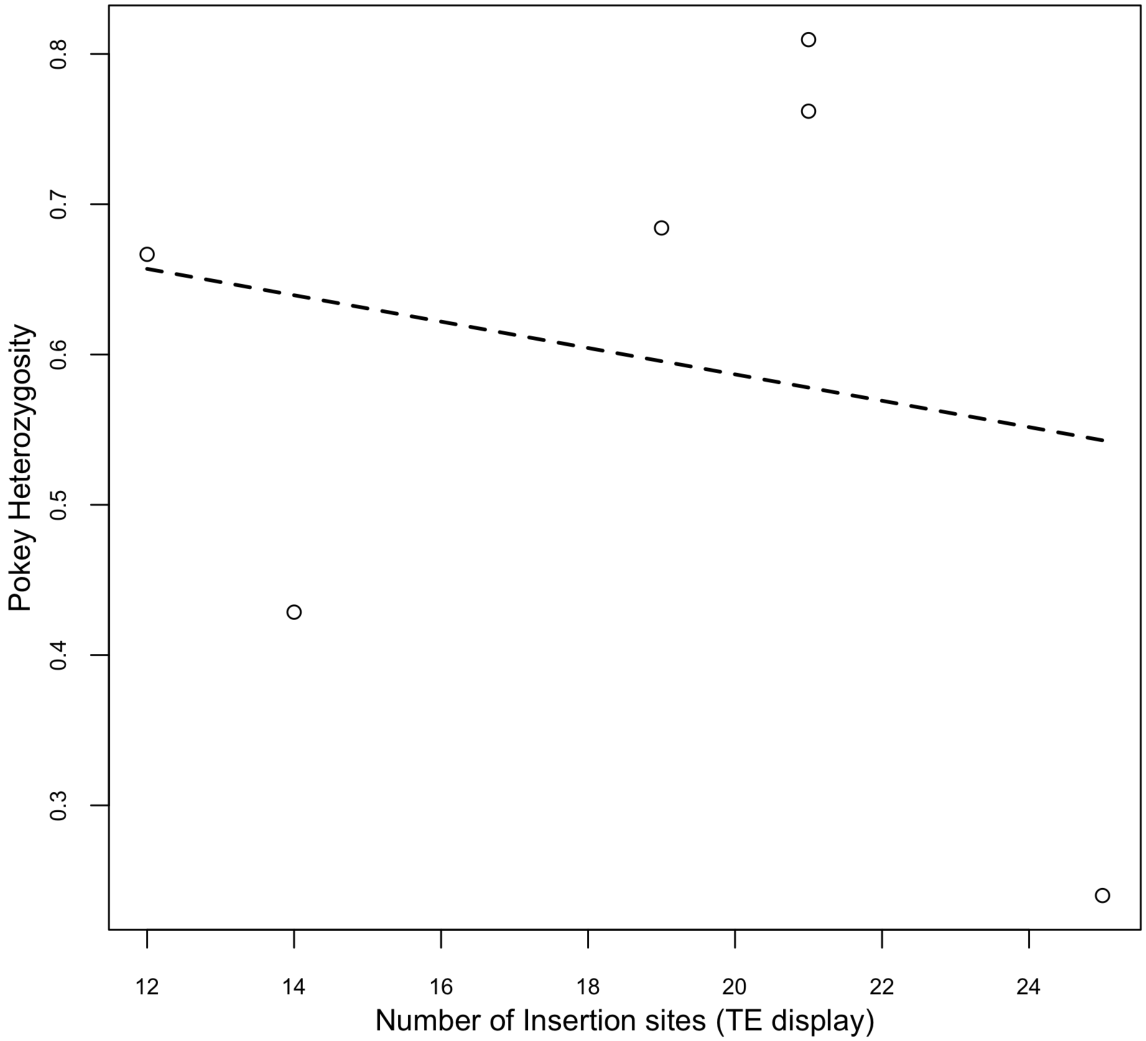

Supplement: Additional file 9 — Correlation between average Pokey insertion site heterozygosity (HgPokey) and the number of Pokey insertions outside rDNA based on TE display analysis of diploid isolates. The dashed line represents the linear regression estimated from the data. [file 1759-8753-5-1-S9.pdf]
