# Supplementary material for: Impact of ploidy level on the distribution of Pokey element insertions in the Daphnia pulex complex
Source: Mob DNA. 2014 Jan 2;5:1. doi: 10.1186/1759-8753-5-1 (PMC3882798; doi:10.1186/1759-8753-5-1)

Additional file 10

Correlation between average *Pokey* insertion site heterozygosity (H_{gPokey}) and heterozygosity (H_{pl}) of their diploid hosts based on microsatellite loci. The dashed line represents the linear regression estimated from the data.

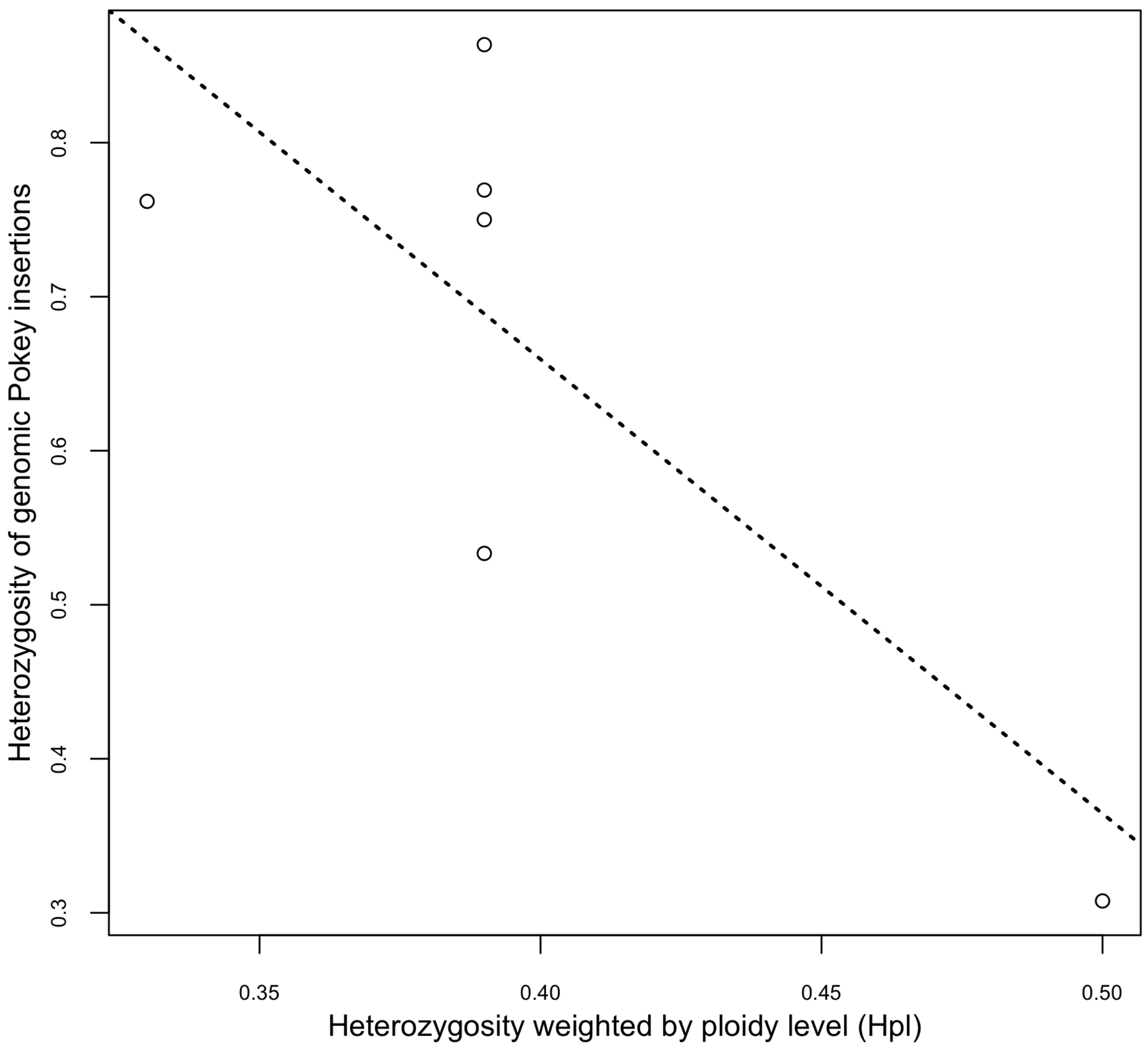

Supplement: Additional file 10 — Correlation between average Pokey insertion site heterozygosity (HgPokey) and heterozygosity (Hpl) of their diploid hosts based on microsatellite loci. The dashed line represents the linear regression estimated from the data. [file 1759-8753-5-1-S10.pdf]
